# Regulating tumor suppressor genes: post-translational modifications

**DOI:** 10.1038/s41392-020-0196-9

**Published:** 2020-06-10

**Authors:** Ling Chen, Shuang Liu, Yongguang Tao

**Affiliations:** 1 Key Laboratory of Carcinogenesis and Cancer Invasion, Ministry of Education, Department of Pathology, Xiangya Hospital, School of Basic Medicine, Central South University, 410078 Changsha, Hunan China; 2 NHC Key Laboratory of Carcinogenesis (Central South University), Cancer Research Institute, Central South University, 410078 Changsha, Hunan China; 3 Department of Oncology, Institute of Medical Sciences, National Clinical Research Center for Geriatric Disorders, Xiangya Hospital, Central South University, 410008 Changsha, Hunan China; 4 Hunan Key Laboratory of Early Diagnosis and Precision Therapy, Department of Thoracic Surgery, Second Xiangya Hospital, Central South University, 410011 Changsha, China

**Keywords:** Epigenetics, Senescence

## Abstract

Tumor suppressor genes cooperate with each other in tumors. Three important tumor suppressor proteins, retinoblastoma (Rb), p53, phosphatase, and tensin homolog deleted on chromosome ten (PTEN) are functionally associated and they regulated by post-translational modification (PTMs) as well. PTMs include phosphorylation, SUMOylation, acetylation, and other novel modifications becoming growing appreciated. Because most of PTMs are reversible, normal cells use them as a switch to control the state of cells being the resting or proliferating, and PTMs also involve in cell survival and cell cycle, which may lead to abnormal proliferation and tumorigenesis. Although a lot of studies focus on the importance of each kind of PTM, further discoveries shows that tumor suppressor genes (TSGs) form a complex “network” by the interaction of modification. Recently, there are several promising strategies for TSGs for they change more frequently than carcinogenic genes in cancers. We here review the necessity, characteristics, and mechanisms of each kind of post-translational modification on Rb, p53, PTEN, and its influence on the precise and selective function. We also discuss the current antitumoral therapies of Rb, p53 and PTEN as predictive, prognostic, and therapeutic target in cancer.

## Background

It has generally acknowledged that cancer is caused by somatic mutations, which is a concept significantly confirmed by demonstrating that cellular proto-oncogenes contribute to carcinogenesis when mutations deregulated or abnormally overexpressed.^1,2^ Our understanding is that many of these genes encode proteins that control cell proliferation, differentiation, and development, while mutations that affect their function constitutively deregulate specific signal pathways, providing some of the clearest insights into how and why abnormal behave of cancer cells happen.^3^ The discovery of dominant “activating” oncogenes has also generated the idea that a unique class of “suppressor genes” may counter their effects and prevent the development of tumors. In fact, experiments about somatic cell fusion or chromosome separation have shown the presence of genes that inhibit tumorigenicity.^4^ Carcinogenesis is a very complicated process, which can be attributed to either mutation of oncogene function or tumor suppressor gene (TSGs).^5^ Our understanding of TSGs mostly comes from the preliminary study of retinoblastoma genes, the first discovery of a TSG, and mutation causes retinoblastoma in children.^6,7^ This is a genetic disease caused by the retinoblastoma susceptibility gene (Rb1) gene inactivation mutation. Compared with the general population, Rb1 gene inactivation mutation increases the risk of retinoblastoma (usually in the eyes) by 10,000 times. These patients also have a high risk of acquiring osteosarcoma and other sarcomas. However, about 60% of retinoblastomas are sporadic (almost in one eye), and these patients have a low risk of other types of cancer.^8^ Therefore, in 1969, the presence of TSGs based on the developmental dynamics of sporadic and hereditary retinoblastoma, which suggested a carcinogenic “2-hit” model, and was eventually accepted and successfully cloned Rb1 in 1986.^9,10^ One of the early famous arguments aimed at the being of TSG was because it is irreconcilable Knudsen’s 2-hit model with Nowell’s cancer clonal evolution model, in which reckoned that cancer is the outcome of cell evolution through continuous clonal selection waves.^1^ It is now supported that for many TSGs, loss of heterozygote function is associated with tumorigenesis by reduced gene dosage and haploinsufficiency.^11,12^ TSGs could be classified into two categories: the one is “gatekeeper” gene and the other is “caretaker” gene.^13^ The gatekeeper gene controls the progress of cells in the growth or division cycle, while the caretaker gene maintains the integrity of the genome.^14^ The difference between these two types of genes is important to the development of therapies. Intuitively, it is likely that inhibiting highly active oncogenes is easier than restoring the function of inactivated TSG. Although they are more difficult to “medicate”, changes in TSG dysfunction are equally important for tumorigenesis. The promising approaches to “medicine” TSG are focus on regulating, inhibiting, or epigenetic silencing of TSG molecules, and closing abnormally activated signaling pathways due to TSG deletion.^15^ TSGs can inhibit or repress cell cycle or promote cell apoptosis. Over the past 30 years, many of these TSGs have been recognized (Table 1). Because they usually only need one functional gene to prevent cancer, the typical TSGs are recessive, and they need two alleles of “second strike” inactivation.^9,16^ Previous studies indicate that only a copy of a TSG is enough to manipulate cell proliferation; in this way, two alleles of a TSG must be consistently inactivated or deleted to bring about tumorigenesis.^17,18^ Therefore, the earliest identification methods relied on genetic methods, biallelic gene inactivation for example, usually in one mutant allele is passed on through the germline and the other is lost somatically. In retrospect, these characteristics define the three basic properties of a “classical” TSGs. First, they are recessive, and then undertake biphasic inactivation in the tumor. Second, the pass on of a single mutated allele benefits the susceptibility of the tumor, since only the other additional mutation is needed for gene function completely lost. Thus, germline mutations may be the root cause of familial cancer syndromes that will inherit. Third, the same gene is often losing activity in sporadic cancers.^19^ At present, TSG, which does not meet the definition of this standard, includes genes that are inactivated by epigenetic silencing rather than deletion. In addition, ubiquitination of proteasome degradation, mis-localization, and abnormal transcriptional regulation are also engaged in the deactivated of TSGs.^20^ Various kinds of cancer, including prostate, breast, glioblastoma, stomach, liver, lung, and leukemia, have abnormal patterns of DNA methylation, including hypermethylation and hypomethylation.^21^ Hypermethylation of CpG islands which are in TSG promoters, such as Braca1, Rb, or p53 promoters, leads to inactivation of each protein, causing cancer.^22^ Two cytosine analogs have been approved by FDA for the treatment of myelodysplastic syndrome (MDS), they are 5-azacytidine/vidaza (AZA) and 5-aza-2′-deoxycytidine/dacogen (DAC).^23^ The second generation of simulated guadecitabine (SGI-110), an active metabolite of gemcitabine, is currently undergoing clinical trials in MDS and acute myeloid leukemia (AML).^24^ Besides, most drugs for cancer are targeted to oncogene, TSGs have difficult to be “drug” treated for they are more likely to change than oncogenes. Nowadays, promising strategies have emerged for TSGs or pathways controlled by these genes.^15^ TSGs nowadays also generally divided into five types: (1) Genes that control cells to enter specific stages of cell cycle;^25^ (2) a signal receptor, a signal transduction gene or a hormone that can inhibit cell proliferation;^26^ (3) Genes that code for checkpoint control proteins trigger cell cycle stagnation when DNA damage or chromosomal defects occur;^27^ (4) Genes that induce apoptosis;^28^ (5) Genes associated with DNA repair.^29^ TSGs have become an important vector response to chemotherapy.^30,31^ TSGs are often affected by mutation or epigenetic disorder in cancer, therefore occurrence and development of all types of cancer along with an important signal molecule in cells.^8,32^ Manipulation of cell survival and death is important to development and growth of organisms.^33^ Activation or inhibition of the cell death is essential for molding and organizing tissues in the process of development organisms.^34^ Signal balance promotes or damages cell survival by impacting on cell aging and various pathologies. Improper cell loss can result in degenerative and autoimmune diseases, and the mutant cells were not eliminated from the constraints of normal cell growth control causes cancer.^35^ Therefore, survive and death signals work co-operational to control cell quality viability.^36,37^

**Table 1:** Selected tumor suppressor genes

Gene	Function	Associated cancer	Others major tumor
p53	Transcription factor	Li-Fraumeni syndrome	>50% of cancers^470,471^
Rb	Transcriptional corepression	Retinoblastoma	Many^114,119^
PTEN	Phosphatase	Cowden syndrome	Glioblastoma, endometrial, thyroid, and prostate cancers^472,473^
RASSF	Transcription factor	Many	Many^474^
ARF	MDM2 antagonist p53 activator	Melanoma	Many^475–477^
APC	Wnt/Wingless signaling	Familial adenomatous polyposis	Colorectal cancer
			Gastro-intesinal tumors^478,479^
ATM	DNA damage sensor (protein kinase)	Ataxia telangiectasia	Lymphoreticular malignancies^480^
CHK2	Protein kinase (G1 checkpoint control)	Li-Fraumeni syndrome	Solid tumors^481,482^
			Carcinomas of the colon, stomach, and endometrium^482,483^
BRCA1, BRCA2	DNA repair	Familial breast and ovarian cancer	Skin cancer, colorectal cancer^484,485^
TSC1,2	GTPase activating protein complex	Tuberous sclerosis	Renal cell carcinoma, angiofibromas^486^
NF1	GTPase activating protein for Ras	Neurofibromatosis	Sarcomas, gliomas^487,488^
LKB1	Serine/threonine kinase	Peutz-Jeghers syndrome (PJS)	Non-small lung cancer (NSCLC), cervical cancer, ovarian cancer, and breast cancer^489^
FOXO3a	Transcription factor	Many	Many^490^

*RASSF* Ras association domain family, *APC* adenomatous polyposis coli, *ARF* ADP-ribosylation factor, *ATM* ataxia telangiectasia mutated, *CHK2* checkpoint kinase 2, *BRCA1* breast cancer 1 protein, *TSC* tuberous sclerosis complex, NF1 neurofibromatosis type 1, *LKB1* the liver kinase B1, *FOXO3a* forkhead box class O3a

Post-translational modifications (PTMs) are key steps in signal transduction of phosphoric acid, acetyl, and glycosyl groups from one protein to another. Because most PTMs are reversible, normal cells use PTMs as a “switch” to decide the cell’s static and proliferative state, which can quickly and strictly regulate cell proliferation. In cancer cells, the oncogene activation and/or inactivation of TSGs supply with ongoing proliferation signals by regulating the diversity of PTMs states of effector proteins involved in cell survival, cell cycle, and proliferation regulation, resulting in abnormal proliferation of cancer cells.^38,39^ PTMs are the core of many cellular signaling events. In addition to a single regulatory PTM, there are some PTMs that work in a coordinated manner. This PTM crosstalk is usually a fine-tuning mechanism that adjusts the cell’s response to small changes in the environment.^40^ Specific protein modification manages almost all cellular physiological processes, such as immune function, as well as the precise location, duration, and intensity of physiological processes to ensure rapid and dynamic cellular responses to extracellular and intracellular stimuli.^41^ Further, PTMs can play as a tight junction (TJ) protein and regulate the function of epithelial barrier.^42^ Compared with transcription or translation regulation, PTMs are fast and dynamic processes, and engaged in the context of barrier maintenance, therefore, PTMs may be essential to work with the altar of environment or external impact. PTMs can regulate formation of membrane-free organelles and supply a potential new treatment strategy for neurodegenerative diseases that cannot be treated at present.^43^

So far, more than 450 unique protein modifications have been found, including phosphorylation, acetylation, ubiquitination, and SUMOylation. These modifications can change the activity, intracellular distribution, protein interaction, and protein life span of the target protein.^44^ Phosphorylation mainly takes place in serine, threonine, and tyrosine residues of the targeted protein.^45^ According to different substrates and phosphorylation sites, protein stability, protein interaction, protein location, and enzyme activity were determined.^46^ Ubiquitination is a well-known post-translational protein modification that manages biological processes, immune responses, apoptosis, and cancer, for example.^47^ As a post-translational protein modification, SUMOylation has attracted more and more attention, for this pathway is necessary to maintain genome integrity, transcriptional regulation, gene expression, and signal transduction in cells.^47^

TSGs work cooperativity in cancers and their function is largely influenced by the posttranslational modification.^15,17^ Ten genes in the human genome are collectively referred to as Ras related domain family (RASSF). RASSF consists of two subclasses: C-RASSF and N-RASSF. N-RASSF and C-RASSF encode Ras related proteins, which are often inhibited by DNA hypermethylation in human cancer. But C-RASSF and N-RASSF are very different. Six C-RASSF proteins are reckoned by a C-terminal coiled-coil motif called the Salvador/RASSF/Hippo domain, while N-RASSF proteins interact with the mammalian Ste20 like kinase, which is the core kinase of the tumor suppressor Hippo pathway.^48^

ADP-ribosylation factor (ARF) plays a crucial role in preventing the development of cancer by regulating cell proliferation, aging, and apoptosis. As a factor inducing aging, the role of ARF as an antitumor factor is closely related to the p53-MDM2 axis, which is an important process to inhibit tumor formation. Although it is generally believed that ARF expression is majorly modulated at the transcriptional level, studies on post-translational regulation of ARF have shown that ARF proteins can be degraded through ubiquitination.^49^

Adenomatous polyposis coli (APC) is considered to be a tumor suppressor gene for colorectal cancer (CRC) and is dysregulated at the germ line and somatic level.^50^ APC activity is related to phosphorylation mediated by CK1 and GSK3β kinase,^51^ which dramatically enhances its affinity for β-catenin to inhibition of Wnt signaling.^52^

MKRN1 plays as an activator of the Wnt/β-catenin signaling pathway by inhibiting APC for MKRN1 is an E3 ligase which can be ubiquitinated APC.^53^

Serine threonine kinase checkpoint kinase 2 (CHK2) is an important DNA damage checkpoint protein for the ATM-p53 signaling pathway. Phosphorylation and ubiquitination are both important post-translational modifications for its function.^54^

Two key factors of TSGs engaged in the homologous recombination (HR) pathway in humans: breast cancer type 1 susceptibility protein (BRCA1) and its obligatory partner BRCA1-associated RING domain protein 1 (BARD1). Mutations in BRCA1 bring about not only familial breast and ovarian cancers but are also the promoters of different kinds of sporadic cancers. BRCA1–BARD1 heterodimers, through their ability of E3 ubiquitin ligase and interact with DNA and DNA damage response factors, benefit to import DNA double-strand breaks, into the HR pathway for repair.^55^ Partner and locator of BRCA2 (PALB2) has become a crucial and versatile participant in genome integrity maintenance. The double allele mutation in PALB2 results in Fanconi anemia (FA) subtype FA-N, while monoallelic mutation is prone to breast and pancreatic cancer.^56^ Regulation of PALB2 involves different post translational modifications of protein, such as phosphorylation and ubiquitination.^57^

Tuberous sclerosis complex (TSC) is an autosomal dominant disease, which is caused by the loss of function mutation of TSC1 or TSC2. It is characterized by a wide range of clinical characteristics in multiple organs such as skin, brain, eyes, lungs, heart, and kidney.^58^ TSC-1 and TSC-2 are tumor suppressors that inhibit cell growth. Mutations in both genes can lead to multiple benign tumors. The products of TSC1 and TSC2 gene form a functional complex with GTP enzyme activating protein (GAP) activity, which has the effect of inhibiting the target of mammalian rapamycin complex 1 (mTORC1), while mTORC1 is constitutively activated in TSC mutant tumor.

Neurofibromatosis type 1 (NF1) is an autosomal dominant genetic disease with an estimated prevalence of 1 in 3000–4000 person. NF1 is characterized by the development of benign tumors in the peripheral nervous system and an enhanced risk of malignancy. The phenotype of NF1 is variable and several organ systems are affected, including bone, skin, iris, and central, and peripheral nervous systems.^59^

The liver kinase B1 (LKB1, encoded by STK11) is a tumor suppressor function as a highly conserved serine/threonine kinase. Phosphorylation is the most common post-translational modification of LBK1 that affects the conformation of LBK1 and creates new surfaces that interact with other proteins. Ubiquitination of proteins is a post-translational modification that, in addition to its well-known functions in protein degradation, is engaged in many other cellular processes, such as activation of the LKB1–AMPK axis.^60^ The location of LKB1 is not limited to plasma membrane, but occurs in nucleus and cytoplasm, which depends on cell type and state, but on C-terminal conserved cysteine 430, LKB1 is farnesylated. Farnesylation is another kind of post-translational modification that mediates a transient membrane connection.^61^ LKB1 is also a target for endogenous neddylation and its endogenous neddylation level is increased in hepatocellular carcinoma (HCC). Neddylation is a post-translational modification that relies on NEDD8 binding to target proteins. Similar to ubiquitination and SUMOylation, neddylation needs E1, E2, and E3 enzymes^62^

The forkhead box class O (FOXO) family is a widely expressed transcription factor that woks in higher organisms. FOXO3a, or FOXO3 or forehead in rhabdomyosarcoma like 1 (FKHRL1), is a member of FOXO3 subfamily, which was first found in human placenta. FOXO3a activity can be modulated by many PTMs, such as phosphorylation, ubiquitination, acetylation, and methylation. Translocation of FOXO3a can be altered by those reversible PTMs, affected its capability of DNA binding affinity, and transcriptional activity patterns at stated gene sites^63^ (Table 1).

Among TSGs, we focus on three important tumor suppressor proteins, Rb, p53, and PTEN, for they are tightly functionally connected and more closely related to post-translational modification. In triple-negative breast cancer (TNBC), Rb and PTEN are often deactivated with p53.^64^ p53, PTEN, and Rb are the most frequently altered TSGs in primary prostate cancer, with abnormal PI3K/AKT, RAS/RAF, and cell cycle signals.^65^ The genomic changes of p53, PTEN, and Rb in early and late prostate cancer (as well as the combined loss of these genes) indicate a poor prognosis.^66^ The changes of p53, Rb, and PTEN have been discovered that they are enriched in drug-resistant diseases, by the genome analysis of metastatic castration resistant tumors.^67^ The formation of glioblastoma requires the disorder of three core pathways: Rb controlled cell cycle progression, p53 signaling pathway, and receptor tyrosine kinase (RTK)/phosphatidylinositol 3′-kinase (PI3K)/AKT axis,^68^ and PTEN negatively mediates the PI3K–AKT–MDM2 pathway that downregulates p53. In addition, p53 also activates PTEN, therefore protecting itself from overly powerful survival signals.^35^ These relationships indicate that proteins induce or inhibit the function of cell death are interconnected.^69,70^ Genetic aberrations influencing the intermediates of these three pathways have been found in almost all glioblastomas.^68^ Rb, p53, or PTEN are TSGs that are found to be inactivated in the tumor matrix of oropharyngeal, breast, and other human cancers.^66^ The mouse model verified the tumor promoting effect of Rb, p53, and PTEN deletion on fibroblasts, which can transform normal fibroblasts into cancer-related fibroblasts (CAFs).^71^ Thus, TSGs are networked to promote normal cell function and eliminate abnormal cells, and this paper attempts to pay more attention to these three tumor suppressor genes.

Moreover, these three tumor suppressor genes, *Rb*, *p53*, and *PTEN* are also deeply influenced by post-translational modifications. In sum, we here explore the influence of those three TSGs, on their functions, as well as new drug targets and strategies for cancer treatment.

### The *Rb* gene, the first tumor suppressor gene and inactivation by multisite phosphorylation

**Fig. 1:**
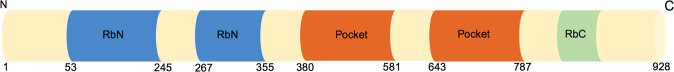
Rb structural domains. Rb structured domains include the N-terminal domain (RbN), the pocket domain, and parts of the C-terminal domain (RbC)

Rb recognition was initially associated with the formation of a rare retinal neoplasm in children, called retinoblastoma.^10,72,73^ Further research shows that changes in the Rb gene or inactivation of Rb protein appeared in many kinds of human cancers, and it is widely believed that Rb inactivation could be one of the most common events in cancer.^74,75^ The functional regulation of Rb includes inhibition of phosphorylation and activation of dephosphorylation events.^76,77^ The Rb phosphorylated by cyclin-dependent kinase (CDK) and checkpoint kinase 2 (CHK2),^78^ while the activation of Rb by dephosphorylation is still rare.^79^ Except a few cases, phosphorylation of Rb brings about inactivation, transcriptional inhibition, and cell cycle progression.^80^ Phosphorylation of Rb regulates the interaction between Rb and other proteins, and this modification usually promotes conformational transition from disordered structure to ordered structure, thus concealing the protein binding surface.^81–83^ Therefore, understanding how Rb is phosphorylated and inactivated requires studying how Rb structure promotes protein–protein interactions and how phosphorylation regulates these interactions.^84^ Rb consists of two independently folded domains and a substantial number of inherently disordered first-order sequences (approximately 33% of the 928 amino acids). The structure of N terminal domain (RbN) and central pocket domain are composed of two helical subdomains (Fig. 1).^85,86^

Rb deletion allows cancer cells to bypass two different barriers in the progression of tumors.^87,88^ Firstly, Rb loss decreased the requirement of amplification of p38 mitogen-activated protein kinase (MAPK) signal when malignant progression. Rb phosphorylated by CDK2 is an effector of p38 mitogen-activated protein kinase (MAPK) signal and a regulator of resisting CDK4 and CDK6 suppression.^89^ Secondly, Rb inactivation relieves the expression of cell state determinants, promotes lineage infidelity, and increases the acquisition of metastasis ability.^90^ The high phosphorylation level of Rb controls its association with early region 2 binding factor (E2F) and depresses its tumor suppressive properties. However, activated Rb can be mono-phosphorylated at any of the 14 CDK phosphorylation sites during G1, and the 14 sites coordinate the interaction of Rb, which endow it with functional specificity.^91^ The mono-phosphorylation of Rb at serine 811 (S811) alters the transcriptional activity of Rb by promoting its binding with nucleosome remodeling and histone deacetylation (NuRD) complex. Mono-phosphorylation of Rb at S811 or threonine (T826) activates the expression of oxidative phosphorylation genes, which increases cell oxygen consumption. The activation signal of Rb might be integrated into a phosphorylation code that controls the different activities of Rb.^91^ The interaction between Rb and nuclear factor-kappa B (NF-kappa B) protein p65 is mainly dependent on the phosphorylation of S249/T252 mediated by CDK4/6 of Rb, and S249/T252 phosphorylated Rb was negatively correlated with programmed death ligand-1 (PD-L1) expression in patient samples, which indicates that hyperphosphorylated Rb-NF-kappa B axis can be used to overcome cancer immune evasion induced by traditional or targeted therapies.^92^ Phosphorylated proteomics data suggest that Rb phosphorylation is associated with reduced proliferation and inhibited apoptosis in colon cancer cells, explaining why this classical tumor suppressor is enrichment in colon cancer and provides a theoretical basis for the application of targeted Rb phosphorylation.^93^ Those results reveal that Rb activation signals can be integrated in a phosphorylation code that will control the diversity of Rb activity,^91^ indicating that phosphorylation of Rb manages interaction with different proteome, chooses different targets, and controls different aspects of Rb function.

### Effects of other post-translation modifications on Rb

Rb is also controlled by other types of post-translation modifications, which may affect Rb in different ways. Oncoproteins binded Rb are often targeted at Rb and degraded by proteasomes during carcinogenesis.^94^ In proteasome, Rb protein is degraded by ubiquitin dependent and non-ubiquitin dependent pathways. Human U3 protein 14a (hUTP14a) interacts with Rb and promotes poly-ubiquitination and turnover of Rb, indicating that nucleolar proteins can be used as nucleolar sensors to directly send nucleolar interruption signals to p53 and Rb, which protect cells from nucleolar damage.^94^ TRIM71, protein kinase A (PKA)-mediated phosphorylation of the E3 ubiquitin ligase, degrades Rb, p53, and antigen peptide-loading complex (PLC) by catalyzing K48 linked polyubiquitination, thus reducing immune monitoring.^95^ HAUSP increases in glioma and regulates Rb, which is by stabilizing effect of MDM2 leading to a decrease in Rb levels in cancer cells.^96^ However, CMV PP71 promotes Rb degradation through non-ubiquitin dependent pathway.^97^ The oncoprotein MDM2, a p53 ubiquitin-E3 ligase that mediates Rb degradation through the ubiquitin-dependent and non-ubiquitin dependent pathways.^98,99^

In the whole cell cycle, Rb is by small ubiquitin like modifier (SUMO)ylated at the early G1 phase,^8,100,101^ which activates Rb phosphorylated in the early G1 phase. The SUMOylation of Rb stimulates its phosphorylation level by recruiting a kinase CDK2 containing SUMO-interaction motif (SIM), resulting in over phosphorylation of Rb and release of E2F-1. On the contrary, the lack of SUMO in Rb led to the decrease of Rb phosphorylation, the CDK2 binding, and E2F-1 isolation.^101^ This suggests that in addition to phosphorylation, SUMOylation is also involved in the regulation of Rb during the cell cycle. SUMO protease SENP1 regulates SUMO1 binding of Rb and lamin A/C. SUMOylation is required for the interaction of these two proteins. Importantly, this SUMO1 dependent complex shelters Rb and Lamin A/C from proteasome degradation. SENP1 regulated Rb desumoylation in cell cycle regulation further deepens understanding of Rb proteasome-dependent degradation.^102^ Therefore, those results present that SUMOylation is a molecular switch controlling phosphorylation and cell cycle regulator function.

Rb can be acetylated and methylated in addition to being phosphorylated, SUMOylated, and ubiquitinated. Rb at Lys873 and Lys874 can be acetylated, resulting in increased their affinity for MDM2, and then reduced phosphorylation of Rb.^103^ DNA damage may lead to Rb acetylation, which engaged in cell differentiation.^104^ Methyltransferase Set7/9 methylate Rb at K810, which has negative effects on Rb phosphorylation and growth of cells.^105^

Given the loss or inactivation of Rb function in most human malignancies, further research is necessary to explore whether PTMs affect the molecular interactions of Rb and mediate Rb’s cell cycle function, as well as the immune function that mediates Rb overlap, or whether it is possible to target various aspects of Rb.

### Targeting the CDK–Rb–E2F axis for cancer treatment

In cancer, cell cycles are frequently activated by interfering with the CDK–Rb–E2F pathway, leading to drug efforts to block the pathway.^75,106^ Kinase inhibitors are the most advanced in drug development, although some compounds that target this pathway are also in different stages of development.^107^ The most promising option among CDK inhibitors is undoubtedly inhibitors of CDK4 and CDK6 (called CDK4/6 inhibitors) and compounds are intended to target the ATP binding sites of the CDK complexes.^108^ More than a decade after Pfizer first synthesized palbociclib in 2001, which is the most advanced component of its kind nowadays.^109–111^ Hypo-phosphorylation of Rb is related to G0/G1 stagnation by inhibiting the activity of E2F transcription factors, while hyper-phosphorylation of Rb promotes E2F release and cell cycle to progress from G0/G1 to S phase,and CDK regulates the hyper-phosphorylation of Rb in the cell cycle.^101^ Therefore, CDK–Rb–E2F axis constructs the core transcriptional mechanism that promotes cell cycle progression, determines the time and fidelity of genome replication, and ensures that genetic material precisely goes through each cell division cycle.^75^ Evaluations of a few small molecules that are highly specific CDK4/6 are under way, besides palbociclib (PD332991) there are ribociclib and abemaciclib, which induces pocket protein hypo-phosphorylation and reactivation, bring about cell cycle arrest in G1.^112–115^ Many clinical trials are under way, with the result being reviewed by several groups, PALOMA-2 is in clinical phase III trial and two other CDK4/6 inhibitors, ribociclib (Novartis) and the other abemaciclib (Eli Lilly) are in clinical trials for breast and other cancers as well^116–119^ (Table 2). In addition, PALOMA-3 was in a randomized, double-blind, placebo-controlled phase III trial, compared the efficacy of palbociclib and fulvestrant (an ER antagonist) for ER+HER2− breast cancer that recurred or progressed during hormone therapy.^120–123^

**Table 2:** Components that are being explored to target Rb, p53 family, and PTEN

Drug target	Drug	Indication
CDK kinase	Palbociclib	Perturbations in CDK4, CDK6^113^
CDK kinase	Ribociclib	Perturbations in CDK4, CDK6^114^
CDK kinase	Abemaciclib	Perturbations in CDK4, CDK6^115^
CDK kinase	PALOMA-1	Perturbations in CDK4, CDK6^118^
CDK kinase	LEE 001	Perturbations in CDK4, CDK6^119,491^
CDK kinase	LY 2835219	Perturbations in CDK4, CDK6^492^
MDM2	Nutlins	Inhibitors of the MDM2–p53 interaction^278,493^
MDM2	RG7112	Inhibitors of the MDM2–p53 interaction^494^
MDM2	RG7388	Inhibitors of the MDM2–p53 interaction^495^
MDM2	SAR405838	Inhibitors of the MDM2–p53 interaction^273^
Mutant p53	PRIMA-1	Conversion mutant p53 to wild-type^496^
Mutant p53	NSC319726	Conversion mutant p53 to wild-type^497^
Mutant p53	STIMA-1	Conversion mutant p53 to wild-type^498^
Mutant p53	SCH529074	Conversion mutant p53 to wild-type^367^
Mutant p53	CP31398	Conversion mutant p53 to wild-type^499,500^
Mutant p53	Zinc	Conversion mutant p53 to wild-type^501^
Mutant p73	RETRA	Inhibition mutant p73 interaction with other protein^502,503^
PI3K	Wortmannin	PIK3CA related excessive growth^423^
AKT	ARQ 092	AKT1-associated Proteus syndrome^422^

Since the Rb gene was isolated in 1986 and the first E2Fs gene was cloned in 1992, we have a deep understanding of the role of CDK–Rb–E2F pathway in cancer. In fact, in almost all human malignant tumors, this pathway is out of control in one way or another, leading this pathway an extremely attractive target for cancer treatment.

### Post translational modification in the non-canonical Rb pathway facilitates histone modification and modulates chromosome structure

**Fig. 2:**
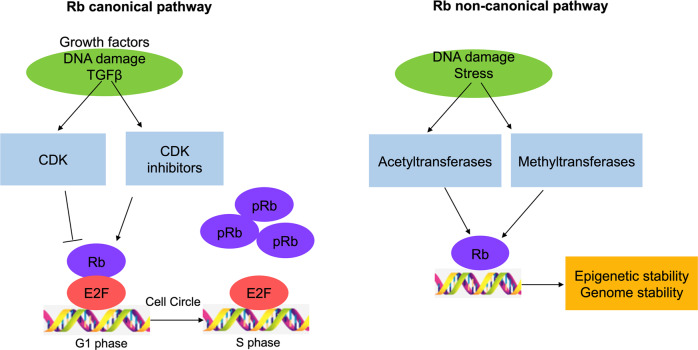
The Rb canonical and non-canonical pathways. Signals of growth factors, DNA damage, and transforming growth factor-β (TGFβ) activate CDKs to phosphorylate and inactivate Rb, whereas CDK inhibitors activates Rb. Inactivation of Rb in the canonical pathway results in transcription of E2F target genes; Stresses and DNA damage stimulates acetylation and methylation of Rb, which can maintain genomic stability by locating DNA break sites and stimulating non-homologous end connections or homologous recombination repair. Rb also recruits enhancers from EZH2 to H3K27me3 to ensure the fidelity of DNA replication and chromosome aggregation

The canonical model of Rb as a TSG developed in the past 30 years is based on the modulation of E2F transcription factors to limit cell cycle progression.^124–126^ In mechanism, non-canonical Rb pathway regulates histone modification and modulates chromosome structure in a way different from cell cycle modulation^127,128^ (Fig. 2).

Chromatin replication maintains Rb-dependent epigenetic markers. An important and indirect mechanism explains the preserve Rb function in the S phase is intrinsic to the chromatin replication. Rb is known to recruit histone methyltransferase enhancer of zeste homolog 2 (EZH2) to guide trimethylhistone H3 lysine 27 (H3K27me3) to deposit and promote octamer-binding protein 4 (OCT4) and Sox2 expressed,^129–131^ which is preserved in the S-phase of cell circle during DNA replication.^132,133^ In turn, the location of H3K27me3 is enhanced in daughter cells via another EZH2 recruitment by Rb in subsequent G1. Thus, mechanisms of maintain epigenetic memory during DNA replication can keep Rb-dependent characteristics without the need for the persistence of the Rb protein presence.^127^ In summary, Rb can be protected by high levels of CDK activity (thus maintaining low phosphorylation levels of Rb), high phosphorylation of Rb preserves function, and Rb relies on histone modification characteristics through the cell cycle. These properties allow Rb to play a role in proliferating cells.^134^

Although the repetitive sequences and sites of DNA damage that is the target of Rb do not appear to require a consistent E2F element (TTTCGCGC), studies have demonstrated that Rb is often engaged in these genomic locations in a E2F1 dependent manner.^129,135^ A mutation in Rb invalidates the interaction between its labeled box domain and E2F1, which has been shown to break the binding of Rb and E2F1 with different types of repetitive sequences.^129,136^

The acetylation and methylation of Rb are caused by DNA damage.^103,105,137,138^ These modifications decrease phosphorylation of Rb by CDK, which further implies that Rb–E2F1 complexes may have a protective effect on CDK activity when it participates in the function of non-homologous end joining (NHEJ) and homologous recombination (HR), for E2F1 is recruited to the sites of DNA double strand break, which is very important for NHEJ, HR^135,139^ (Fig. 2). Hyperphosphorylated Rb also interacts with E2F1 when DNA breaks.^140,141^ These results further suggest that Rb–E2F1 interaction is not sensitive to CDK activity and therefore, these mechanisms of epigenetic and genomic stability depending on Rb–E2F1 are not related to CDK and belong to the non-canonical functions of Rb.

Finally, the multifunctional nature of Rb makes it a key target in many cancer-associated environments. Further, the trans-differentiation phenotype about recurrent cancer from a series of molecular targeted therapies shows that Rb loss is related to acquired treatment resistance, and its pathway is beyond the control of cell cycle. Understanding how Rb loss leads to drug resistance is critical to realizing the function of these targeted molecules.^127^ The loss of Rb in both regulatory pathways in cancer may produce a powerful synergistic cancer promotion combination. These functions of Rb are significant for chemotherapy response and drug resistance of targeted anticancer drugs. This view provides a framework for Rb research in future basic and clinical research.

### Tumor suppressor p53: determinants of its post-translational modifications

Transcription factors (TFs) are always activated through two main mechanisms: (i) the TF levels are increased in the nucleus, or (ii) via post-translational modifications (PTMs).^142^ Tumor protein p53, a TF, is encoded by homologous genes in different organisms, and it is crucial in multiple organisms.^143–145^ p53 is a short-lived protein because of its rapid proteasomal degradation, and it controls the cellular response to different stress signals;^146,147^ therefore, p53 undergoes a variety post-translational modifications following genotoxic stress, leading to enhanced protein stability and translocation to the nucleus.^148–151^ It is well accepted that protein modifications play a significant role in p53 regulation, whose functions vary from regulating p53 stability and localization, to controlling cell proliferation, and cell death.^145^ Post-transcriptional modifications of p53 occur at approximately 50 sites on the peptide, and include phosphorylation, acetylation, mono- and dimethylation, glycosylation, ubiquitination, neddylation, sumoylation, and poly-ribosylation.^152^ Many post-translational modifications occur with or without genotoxic pressure and are relatively independent of each other. Less is known about a possible direct connection between chromatin modification and post-translational modifications. p53 also plays a crucial role in regulating the epigenetic changes that occur in cells due to cross-talk between p53 associated with its modifications.^153,154^ In addition to the role of chromatin remodeling proteins in metabolism and ferroptosis,^155–157^ we have suggested that these proteins may also have post-translational modification functions.^151^

### Phosphorylation of p53 is a critical modification guiding its regulation of apoptotic cell death

Human p53 contains serine (S) and threonine (T) phosphorylation sites across the entire protein, but they are enriched in the transcriptional activation area of the N-terminal domain and the regulatory region of the C-terminal domain.^158^ Some stimuli, including genotoxic stress (DNA damage-inducing agents) or glucose deprivation, induce many reversible PTMs of p53.^159,160^

The phosphorylation of p53 two transactivation domains (TAD) at serine 15 is the initially activated phosphorylation site, and it is phosphorylated by both the ataxia telangiectasia mutated gene (ATM) and ataxia-telangiectasia-mutated-and-Rad3-related kinase (ATR) protein kinases,^161–163^ phosphorylation also can stimulate the association between p53 and histone/lysine acetyltransferase (HATS),^164^ which is quite crucial for the stability and activation of p53. The activation of ATM leads to the phosphorylation of a number of substrates, such as casein kinase (CK1), checkpoint kinase 1 (Chk2), and p53, mediating the effects of ATM on DNA repair, cell-cycle arrest, apoptosis, and other downstream processes. In addition, ATM depleted and p53 mutation are usually mutually exclusive, which shows that these proteins are the same in promoting the survival of cancer cells.^165^ The phosphorylation of Ser15 also triggers a series of other p53 phosphorylation events that contribute to p53 induction and activation, suggesting that Ser15 phosphorylation is a key point in p53 activation.^162,166^ It was reported that phosphorylation of Ser15 led to the dissociation of MDM2 from p53, which increases the stability of p53.^167^ Ser15 can also be phosphorylated via the AMP-activated protein kinase (AMPK) pathway, which is mediated by glucose-dependent cell cycle arrest at G1/S.^168,169^ Further, both IR and UV light can induce phosphorylation of p53 on Ser-20, for ATM and ATR can phosphorylate p53 on Ser-20, which mediates stabilization of human p53 in response to DNA damage.^170^

In addition, p53 function altars from “arrestor” and “repairer” to “killer” depending on many post-translational amino-terminal phosphorylation of p53. The function of Ser46 phosphorylation in p53 is closely related to the killer function of p53 bringing about apoptosis and can be phosphorylated by a number of candidate kinases, such as homeodomain-interacting protein kinase 2 (HIPK2), p38 and dual specificity tyrosine-phosphorylation-regulated kinase 2 (DYRK2).^171–173^

The interactions between p53 and MDM2 or p300/CBP are regulated by various phosphorylation events in the amino terminus of p53, which leads to the simultaneous binding of one monomer of p300/CBP to tetrameric p53 to mediate p53-dependent transactivation in response to genotoxic stress.^174,175^ p53 cooperates with the apoptosis stimulating proteins of p53 (ASPP) proteins being able to bind and work p300 together, selectively regulating the apoptotic function of p53.^176,177^ The Ser 6 and 9 sites were initially thought to be phosphorylation sites of the protein kinase CK1 family members, CK1d and CK11.^178^ The function of Ser 6 and Ser 9 phosphorylation in p53 is to integrate TGF-beta and FGF-signaling by inducing the interaction between p53 and Smad, which may be important in tumourigenesis and metastatic progression.^179^ Smad plays as crucial platforms in mutant-p53/p63 protein complex, and when Ras signaling accelerates mutant-p53 phosphorylation, mutant p53 and Smad interrupt p63 to form a ternary complex, in doing so, the p63 transcriptional functions are antagonized.^179^ The role of amino-terminal phosphorylation is to regulate the interaction between p53 and its inhibitor, MDM2, or its coactivators p300/CBP, and growth factor-mediated phosphorylation coordinates physiological and developmental signals.^152,180^ Those results suggest that transcriptional coactivator p300/CBP is an important player in activating p53.

### Acetylation of p53 engaged in the fine tuning of cellular responses to DNA damage and genotoxic stress

Acetylation of p53 is an important form of post-translational modification that is essential for its activation, and the acetylation occurs via a reversible enzymatic process.^181–183^ Both acetylation and ubiquitination can modify the same lysine residues at the C terminus of p53 (similar to neddylation and methylation), and these modifications are mutually exclusive and have different effects on p53 regulation.^184–188^

Six p53 lysine (K) residues within the C-terminal regulatory domain (K370, K372, K373, K381, K382, and K386) can be targeted by MDM2.^158^ These modifications lead to activation of the transcriptional activation activity of p53 and increase its stability. CBP/p300 are transcriptional coactivator proteins that play a dual role in regulating p53 function. For one thing, an interaction between p300 and either p53 or E2F1 has a significant impact on early cell cycle progression, suggesting that a critical role for p300 in cooperation with the pathways of growth arrest regulated by E2F and p53.^189^ For another, they facilitate the ubiquitination of p53 by MDM2, which decreases p53 levels in the presence of genotoxic stress.^190^ They also protect p53 from degradation by acetylating the p53 carboxyl terminus, which contains targets for ubiquitination. K320, present in the tetramerization domain, can be acetylated by PCAF after DNA damage, and this acetylation is beneficial for cell survival as it boosts the expression of p53-controlled cell cycle arrest target genes, such as cyclin-dependent kinase inhibitor 1A (CDKN1A, commonly known as p21).^191,192^

Unique to these residues, K120-acetylated p53 accumulates at mitochondria, which is thought to negatively regulate apoptosis by affecting the Bak/Mcl-1 interaction.^193^ In the p53 DNA-binding domain, K120 also can be acetylated by human males absent on the first (hMOF) and Tip60, which is quite essential for the activation of target genes connected to apoptosis but not to those involved in cell cycle arrest.^194^ In addition, K120 and K164 are present in the p53 DNA-binding domain, which is the most common region for p53 mutations in malignant solid tumors, indicating that they might be connected with p53 function in cancer. A K120 mutation was found in Ewing’s Sarcoma and esophageal SCC cells, while a mutation in K164 was discovered in glioblastoma and bladder carcinoma.^188,195^ These data indicate the key role of p53 acetylation in tumor suppressive activity.

### p53 methylation contributes to its tumor suppressor activity

Lysine (K) and arginine (R) residues in p53 can be methylated, and a growing number of studies in recent years have shown that p53 methylation takes place during the DNA damage response.^196–198^ Methylation of lysine and arginine residues in histones has long been known to impact chromatin structure and gene expression.^199^ In recent years, the methylation of p53 has emerged as an important modification that affects its function in various processes, such as cell cycle arrest, DNA repair, senescence, apoptosis, and tumourigenesis.^199^ Whether p53 is activated or depressed depends on the location of the modification and the number of methyl groups attached.^200^ Protein arginine *N*-methyl transferase 5 (PRMT5) was first shown to methylate p53 at several arginine residues (R333, R335, and R337) in the tetramerization domain,^196^ which specifically controls the functions of p53 in cell cycle arrest and is suggested to inactivate p53 during lymphomagenesis.^201,202^ There are three different lysine methyl transferases (KMTs) that could mono-methylate p53, and there are at least two KMTs could di-methylate p53.^203^

Monomethylation of p53 by SET and MYND domain-containing protein 2 (SMYD2) at K370, which was shown to repress p53-mediated transactivation, decreases the binding of p53 to the promoters of its target genes, such as p21.^204^ Monomethylation at K372 by SET7/9 boosts the activation of p53 downstream target genes, but monomethylation of K370 by SET8 inhibits p53 transcriptional activity.^205,206^ In addition, a second methyl group can be conjugated to p53 to form K370me2, which then promotes p53 function via stimulating its binding to the Tudor-domain-containing reader, p53 binding protein 1(p53BP1). Like K370Me2, K382Me2 has also been shown to be related to the stabilization and activation of p53. Interestingly, lysine-specific demethylase 1 (LSD1) selectively wipes off this second methyl group, thus inhibiting p53 function by interrupting the association of p53 with 53BP1, which contributes to these effects.^207,208^ Thus, p53 contributes to keep DNA methylation homeostasis and clone homogeneity, which may benefit to its anti-cancer activity.

### p53 SUMOylation regulates p53 localization

The tumor suppressor p53 experience dynamic nuclear output, because its tetramer domain contains a nuclear export signal (NES) domain full of leucine.^209^ The N-terminal transactivation domain of p53 seems containing another NES, in which phosphorylation blocks the nuclear output of p53, bring about its nuclear accumulation.^210^ SUMOylation occurs at K386 of p53 and SUMO-1, SUMO-2, or SUMO-3 that accelerates the output of the p53 from nucleus.^211–213^ p53 in the nucleus not only promotes the expression of pro-apoptotic genes but also prevents cell death by increasing p21 expression.^214^ Most p53 anti-apoptotic functions happen in the nucleus, especially under resting conditions.^214,215^ p53 is normally SUMOylated at a single site, K386, by the protein inhibitor of activated stat (PIAS) family members and Topors.^216,217^ SUMO E3 ligase PIASy and lysine acetyltransferase Tip60 involved in p53-mediated autophagy. The combination of PAISy to p53 and then PAISy activated Tip60 resulted K386 sumoylation and K120 acetylation of p53, respectively. Although these two modifications are not interdependent, they together act as “binary death signals” and promote the accumulation of p53 cytoplasm and the execution of PUMA mediated autophagy.^218^ When the COOH-terminal nuclear export signal of p53 is masked by its unmodified C-terminal region, it remains in the nucleus. Moreover, the SUMOylation of p53 releases it from the chromosomal region maintenance 1 (CRM1) Huntington-EF3-PP2A subunit-HEAT9 loop to disassemble the transporting complex and promote the translocation of p53 to the cytoplasm.^219^ Thus, the nuclear export of p53 can facilitate cellular proliferation through the loss of its antigrowth function. Cytosolic p53 performs a non-transcriptional function by interacting with, and then counteracting, the anti-apoptotic function of Bcl (B cell lymphoma/leukemia)-2.^220^ In addition, p53-Bcl-2 binding depends on p53 SUMOylation,^221^ and a lot of cytoplasmic p53 localization is clinically associated with poor prognosis and disease progression to hormone-resistance status.^222^

### Ubiquitination/proteasome-dependent protein degradation is important for rapid signal transduction

**Fig. 3:**
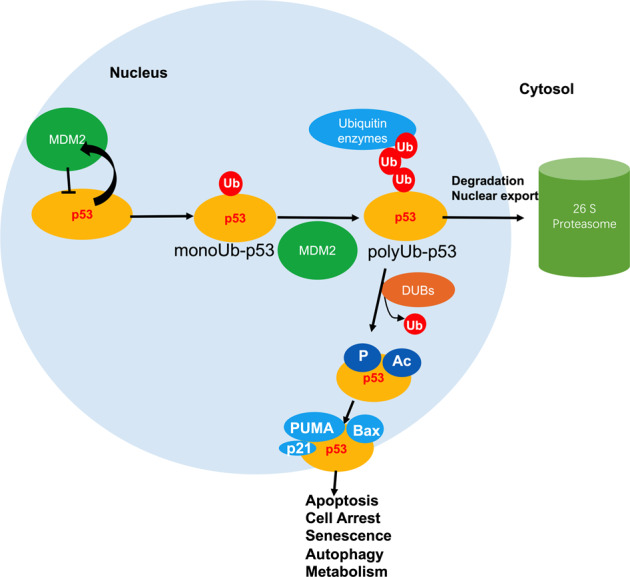
Ubiquitinated and de-ubiquitinated p53 functions and pathways. p53 is degraded after MDM2-mediated ubiquitination, and other DUBs stabilize p53 by eliminating ubiquitin from p53. Under normal conditions, MDM2, a target of p53, inhibits p53 activity by forming a p53/MDM2 auto-regulatory feedback loop. Furthermore, MDM2 can bind to p53 and control p53 monoubiquitination, leading to the nuclear export of p53. Other E3 ligases further promote p53 poly-ubiquitination and 26S proteasomal degradation in the cytoplasm. Upon DNA damage, DUBs localize to the nucleus and de-ubiquitinate p53 to alter its stability, thus boosting p53 activation. Consequently, p53 is activated through various kinase or acetyltransferases, after which it binds to its transcriptional targets, including p21, p53 upregulated modulator of apoptosis (PUMA), Bax and Noxa, for example. Ub ubiquitin, DUBs de-ubiquitinating enzymes

Ubiquitin is a highly conserved, stable, small molecule protein with 76 amino acid residues.^223^ The ubiquitin-proteasome system (USP) depends on the small polypeptide ubiquitin and is a delicate process requiring of three classes enzymes: a ubiquitin-activating enzyme (E1), a ubiquitin-conjugating enzyme (E2) and a unique ubiquitin ligase (E3).^224^ Consequently, ubiquitination includes three main steps: activation, conjugation, and ligation by E1s, E2s, and E3s, respectively.^225,226^ Ubiquitin conjugation to proteins can control various biochemical reactions, such as precursor protein maturation, degradation of unneeded proteins, and protein turnover.^227^ Ubiquitination begins with the attachment of a ubiquitin molecule to Lys residue.^228^The key characteristic of ubiquitin is its seven Lys residues can be ubiquitinated to produce ubiquitin chains linked to isopeptides. When a ubiquitin is connected to the N-terminal of the second ubiquitin, the eighth chain type, MET1 chain or “linear” chain, is generated.^229–232^ Consecutively assembled ubiquitin molecules generate a poly-ubiquitin chain that is formed on the target proteins and is the degradation signal recognized by the 26S proteasome subunit (Fig. 3).^233,234^ Subsequently, the protein substrate would be degraded into shorter peptides, resulting in the release and reuse of ubiquitin.^235^ In addition to ubiquitin, Small Ubiquitin like MOdifier (SUMO), NEDD8 (downregulated protein 8 of neural precursor cell expression), ISG15 (interferon stimulation gene 15) or FAT10 (HLA-F adjacent transcript 10) can also be coupled to the target proteins. These peptides are classified into the ubiquitin like protein (UBL) family and have similar structure with ubiquitin.^236^

Protein modifications can be achieved by either a ubiquitin molecule (mono-ubiquitination) or by a chain of ubiquitin (poly-ubiquitination).^237–239^ Polyubiquitination, in which four or more ubiquitin monomers are bound to a substrate, occurs mostly on K48 and K29 and is regarded as a “molecular kiss of death” as it is associated with proteasome-dependent degradation.^240–242^ K63-linked ubiquitination is associated with aggregate formation, lysosomal degradation, and protein interactions.^243–246^ Monoubiquitination and multiple monoubiquitinations are involved in various processes, including trafficking, inflammation, DNA repair, and histone regulation.^247,248^ Therefore, ubiquitination regulates proteins in several ways: it can alter their location in cells, impact their activity, control their degradation by the proteasome, and stimulate or prevent protein interactions.^249,250^ Recently, more and more attention has been paid to the regulation of transcription factor function by ubiquitination. The primary sites for p53 ubiquitination are located at its C terminus, where acetylation takes place during times of cell stress and functions to block protein degradation, maintaining p53 stability.^235^

### MDM2 is a key negative regulator of p53

Mouse two-minute two (MDM2) is an oncogene that accelerates cell growth, survival, invasion, and contributes to therapeutic resistance, and the most well-known function of MDM2 is that it works as an E3 ubiquitin ligase. Physiologically, MDM2 antagonizes tumor suppressor p53.^251^ MDM2 inhibits the stability of p53 by ubiquitination. In addition, p53 inactivation was managed by MDM2 and in turn, MDM2 affected the subcellular localization of p53. MDM2 is often overexpressed in some human and mouse malignant tumors.^252^

**Fig. 4:**
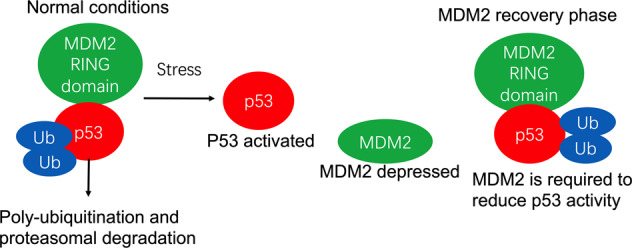
Autoregulatory loop of p53 and MDM2. The RING finger domain of MDM2 is involved in p53 ubiquitination and proteasome-mediated degradation, and, therefore, MDM2 maintains appropriately low p53 levels under unstressed conditions. Activated p53 transcribes MDM2 mRNA and increases the level of MDM2 protein, which in turn reduces p53 activity

MDM2, first recognized E3 ligase to regulate p53 stability, contains a RING finger domain and interacts with Ubc5 (E2 ubiquitin-conjugation enzyme), which can ubiquitinate p53 both in vitro and in vivo and, via the proteasome system, is a crucial negative regulator of p53.^253–255^ The RING finger domain of MDM2 includes a sequence that prevents the activity of E3 ubiquitin-protein ligase;^256,257^ therefore, MDM2 can regulate its own levels via auto-ubiquitination.^258,259^ CBP/p300 and MDM2 target six lysine residues (K370, K372, K373, K381, K382, and K386) in the C-terminal regulatory domain respectively for acetylation and ubiquitination,^260^ which are essential for the nuclear export of p53. MDM2 is a negative regulator of p53 and can effectively inhibit p53 acetylation mediated by p300/CBP in vivo and in vitro. The suppress activity of MDM2 on p53 acetylation was also eliminated by the tumor suppressor p19 (ARF), suggesting that the regulation of acetylation is an important part of the feedback loop of p53-MDM2-p19 (ARF).^261^ The MDM2 oncoprotein is overexpressed in many types of human cancers and is a critical component of the p53 pathway.^262,263^ MDM2 targets p53 for ubiquitination, and for proteasome-mediated degradation, and it maintains an appropriately low level of p53 under unstressed cell conditions.^264^ MDM2 directly decreases the transcriptional activity of p53 by binding to its N-terminal transactivation domain (TAD).^265^ When MDM2 is overexpressed, there is a loss of p53 activity, and cells acquire limitless replicative potential. Further, MDM2 mediates the nuclear export of p53.^266^ Moreover, when p53 is ubiquitinated by MDM2, it cannot be acetylated by p300/CBP, and, therefore, rapid proteasome-mediated degradation takes place.^261^ As MDM2 is transcriptionally induced in a p53-dependent manner, the two proteins make an elegant feedback loop (Fig. 4).^267^ When modifications occur on MDM2, the direct interaction between p53 and MDM2 is broken, such as during a DNA damage event in which MDM2 is phosphorylated at serine 395.^268^

Notably, low level of MDM2 activity induces p53 mono-ubiquitination and nuclear output, while high level of MDM2 activity promotes p53 polyubiquitination and nuclear degradation. In summary, MDM2 suppresses p53 in two ways: (i) MDM2 works as an E3 ligase to ubiquitinate p53, thus stimulating its degradation by the proteasomal pathway, and (ii) MDM2 inhibits p53 transcriptional activation by binding to it (Fig. 4).^269^ Therefore, there are two ways to increase the stability of p53: one is to downregulate the expression of MDM2, the other is to regulate the intracellular localization of MDM2 or p53.^270^ Activation of p53 results in its uncoupling from MDM2 and the related proteins, such as MDM4, which principally inhibits p53-dependent transactivation.^271^ Post-translational modification of p53 plays an important role in this process, at least in the DNA damage response. MDM2 is a key mediator of the different stress pathways that impact p53.^272^

It has been suggested a new cancer treatment strategy is that the small molecular inhibitors intended to block the interaction of MDM2-p53 may effectively treat human cancers that remain wild-type p53 through reactivating the anticancer function of p53.^273–277^ After two decades of efforts, many nonpeptide, small molecule inhibitors (MDM2 inhibitors) with unique structure and strong efficacy of MDM2–p53 interaction interrupted have been developed, and Nutlins is the first effective and specific small molecule inhibitor which interrupts MDM2–p53 interaction.^278^ At present, at least seven of these compounds have entered human clinical trials as novel anticancer drugs (Table 2).^273^

Although MDM2 plays a key role in regulating p53 levels and functions, p53 activity is controlled and fine-managed across a wider range of regulators by multiple mechanisms-monitored PTMs.

### Others factors that ubiquitinate p53

p53-induced RING-H2 (Pirh2) is an E3 ligase that has been reported to target p53 for polyubiquitylation and degradation.^279^ Similar to MDM2, Pirh2 is also a transcriptional target of p53, and its transcription is increased in response to DNA damage.^280^ Thus, Pirh2 takes part in an autoregulatory feedback loop that mediates p53 function. Interestingly, there are several differences between MDM2 and Pirh2. For example, phosphorylation of Pirh2 can bring about its own inactivation. In addition, MDM2 mainly degrades p53 in unstressed cells, but Pirh2 is capable of degrading p53 after DNA damage.^268,281^ Furthermore, Pirh2 can regulate the stability of p73, a p53 family member, but MDM2 cannot.^282^ Thus, it is possible that MDM2 specifically polyubiquitinates and degrades p53, whereas Pirh2 can control the protein stability of other p53 family members. Moreover, Pirh2 interacts with p53 and regulates its polyubiquitylation in association with the E2 ligase ubcH5b, independent of MDM2. Further, Pirh2 preferentially binds to and degrades p53 in its tetrameric active form, but not its monomeric form.^283^ These data confirm that Pirh2 is a novel tumor suppressor associated with regulation of p53 and MDM2.

Constitutively photomorphogenic 1 (COP1), an E3 ubiquitin ligase, has been regarded as a direct ubiquitin ligase for p53.^284,285^ COP1 is also a p53-inducible gene (a p53-responsive element exists in the COP1 gene promoter region), and it can ubiquitinate and degrade p53 independently of MDM2, which is necessary for p53 turnover in normal and cancer cells.^284^ Furthermore, in cancers that involve wild-type p53, the expression of COP1 is associated with a significant reduction in the steady state p53 protein levels and with attenuation of the downstream p53 target gene^285–287^; therefore, COP1 inhibits p53-mediated G1 arrest, which is important in cell survival, development, and cell growth. In addition, degradation of p53 by COP1 is impaired upon DNA damage, resulting in p53 stabilization and activation.^288^ The results showed that COP1 was an important negative regulator of p53 and a new pathway for keeping low p53 protein levels in non-stressed cells.

ARF-binding protein 1 (ARF-BP1, HUWE1) is a HECT domain-containing E3 ligase that regulates p53 levels to induce tumor suppression via the stabilization of p53 and the activation of apoptosis.^289–291^ ARF-BP1 contains a ubiquitin-associated domain (UBA, 1318-54), a WWE domain (1612-92), and a HECT domain in the C-terminal sequences (4036-4734).^289^ The UBA domain is a small motif shown in various proteins to be related to the ubiquitination pathway.^292^ ARF-BP1 is a primary binding partner of ARF in cells without p53. Interestingly, ARF effectively represses ARF-BP1-regulated p53 ubiquitination, and it also contributes to the neutralization of ARF-BP1’s p53-independent anti-proliferative effect. In addition, the N-terminal region of ARF showed the strongest inhibition of ARF-BP1-mediated p53 ubiquitination; however, the C-terminal region displays little effect. Therefore, ARF-BP1 plays a crucial role in ARF-mediated p53 stabilization in an MDM2-independent manner.^289^

Trim24 was identified as a member of family of TRIM/RBCC family of proteins, which contain a conserved amino-terminal tripartite motif consisting of a RING domain, B-box zinc fingers, a coiled-coil region, and carboxy-terminal domains.^293,294^ Therefore, Trim24 is an E3-ubiquitin ligase that negatively regulates p53 via ubiquitination through its RING domain to promote proteasome-mediated degradation.^295,296^ Trim24 interacts with phosphorylated p53 to stimulate its degradation. Furthermore, Trim24 is phosphorylated at S768 in response to DNA damage by ATM, which destabilizes Trim24 and interrupts the Trim24–p53 interaction.^296^ However, DNA-damage-activated p53 induces Trim24 transcription via an interaction with p53 response elements. Similar to MDM2, Trim24 controls p53 levels in an autoregulatory feedback loop.^297^ However, unlike MDM2, Trim24 also terminates the activated p53-regulated response upon DNA damage.^296^ p53 is ubiquitinated and negative regulated by Trim24, which indicated that Trim24 is a therapeutic target for p53 to restore tumor inhibition.

Synoviolin, a component of the ER-associated degradation (ERAD) complex, is an E3 ubiquitin ligase that targets p53, and it is engaged in endoplasmic reticulum related degradation, an ATP-dependent ubiquitin-proteasome degradation process that reduces the burden on the ER.^298,299^ Synoviolin sets p53 apart in the cytoplasm and negatively regulates, for example, its protein level and functions, transcription, and cell cycle regulation.^300^ Interesting, the regulation of p53 by synoviolin is irrelevant to the other E3 ubiquitin ligase-formed autoregulatory feedback loops, such as those involving MDM2, Pirh2, and Cop1.^300^ Combined with the antiapoptotic properties of synoviolin previously elucidated in vivo and in vitro studies, its new role in p53 regulation may supply new ideas for studying the pathogenesis of proliferative diseases.

Topoisomerase I-binding protein (Topors) contains an N-terminal C3H4-type RING domain that is similar to the RING domains in E3 ligases, and it contains both ubiquitin-E3 and SUMO-E3 ligase activity.^301,302^ Human Topors, which was originally regarded as a p53-binding protein and functions as an E3 ubiquitin ligase for p53, leads to the degradation of p53.^303^

The caspase 8/10-associated RING proteins (CARPs), CARP1 and CARP2, act as RING-domain E3 ligases that target apical caspases for proteasome-mediated degradation.^304^ In addition to apical caspases, CARPs, which are overexpressed in cancer, physically interact with and target p53 or phospho-p53 for ubiquitination and degradation with or without MDM2. Unlike other E3 ligases, CARPs can ubiquitinate DNA damaged-mediated phospho-p53 at serine 15 or 30.^305,306^

Human ubiquitination factor E4B (UBE4B) is a human mammalian homolog of the Ufd2 protein found in *S. cerevisiae*. Yeast Ufd2 is engaged in the Ufd pathway, which is a proteolytic pathway that recognizes ubiquitin as a degradation signal.^307^ Yeast Ufd2 belongs to a new class of ubiquitination enzyme, E4 (a novel ubiquitin chain assembly factor) and is required for ubiquitin chain assembly.^307^ Mouse UBE4B regulates ubiquitination as a companion to E1 and E2, and independent of the E3 components. UBE4B physically associates with p53 and MDM2,and then promotes p53 polyubiquitination, which results in p53 degradation, thus inhibiting p53-mediated transactivation and apoptosis.^308^

p300 and CREB-binding (CBP) were regarded as multifunctional modulators of p53 through their acetylase and poly-ubiquitin ligase (E4) activities.^309^ p300 and CBP were revealed to be required for endogenous p53 polyubiquitination and rapid turnover in normal cells.^310^ Interestingly, the ubiquitin ligase activity of p300/CBP is present only in nuclear extracts and not cytoplasmic extracts. In accordance to its E3/E4 activity, CBP specifically destabilizes cytoplasmic, but not nuclear p53.^311^ In addition, p53 turnover is observed in p300-deficient or CBP-deficient cells via polyubiquitination of mono-p53. Furthermore, p300 exhibits its E3/E4 activity within its N terminus.^190^ Similar to p300, CBP contains an E3 activity in its N terminus and shows E4 activity towards p53 in vitro.^312^ Therefore, the E4 activity of cytoplasmic p300/CBP destabilizes p53 by ubiquitinating it, while physically distinct p300/CBP activities in the nucleus, such as p53 acetylation, activates p53.^311^

E4F transcription factor 1 (E4F1) is a zinc-finger-containing protein identified as an atypical ubiquitin E3 ligase for p53 by activation oligo-ubiquitylation on p53 lysine residues that are different from the targets of MDM2.^313^ E4F1 physically interacts with p53,^314^ and then conjugates Ub to p53 that is bound to chromatin, a modification that coincides with the stimulation of a p53 transcriptional program that is engaged only to control cell cycle arrest and not apoptosis. E4F1-mediated modification p53 plays a crucial role in the cellular life-or-death decision.^313^

Ubc13 is an E2 ubiquitin-conjugating enzyme, but it increases p53 stability by interrupting K63-dependent ubiquitination of p53, which decreases MDM2-dependent polyubiquitination of p53.^315^ However, Ubc13 increases p53 stability but prevents its tetramerization and increases its location to cytoplasm, which attenuates p53 transcriptional activity.^315,316^ Like MDM2, p53 activation induces the expression of Ubc13 in response to DNA damage, suggesting a feedback loop between Ubc13 and p53. Ubc13 interaction with p53 requires an intact p53 C-terminal domain, and this interaction negatively effects the tetramerization of p53. However, Ubc13 is not capable of contributing to p53 monomerization in response to DNA damage.^316^

LINK-A expression increased the degradation of K48 polyubiquitination-mediated endogenous tumor suppressors Rb and p53, which inhibits immune sensitization of breast tumors.^95^

Thus, p53 are modulated at the level of gene expression and post-translation modification, and at the level of protein stability through ubiquitin proteasome pathway. In the past 20 years, many ubiquitin E3 ligases have been found to promote the degradation of p53 directly or indirectly in vitro and in vivo.

### De-ubiquitinating enzymes (DUBs) eliminate ubiquitin from p53

Ubiquitination governs the division, differentiation, and survival of eukaryotic cells. Ubiquitin system is a powerful signal network by consist with multiple E3 ligases (Writers), ubiquitin binding moleculars (Readers) and de-ubiquitylases (erasers) with different functions. From yeast to human, ubiquitin system is used in a similar way.^317^ De-ubiquitinating enzymes (DUBs) are a group of proteins engaged in the ubiquitin-proteasome system.^289^ The major function of DUBs is to process and recycle ubiquitin; therefore, DUBs reverse ubiquitination of specific substrate proteins, similar to the reversal of protein phosphorylation by phosphatases.^149,284,318^ There are several possible reasons why multiple DUBs are needed to regulate p53 stability and activity. First, different DUBs regulate the p53 pathway when confronted with different cellular stresses; second, different DUBs function in different cellular compartments; and last, since p53 is ubiquitinated by many E3 ligases, DUBs are needed to counteract p53 ubiquitination.^150,318,319^ After p53 is targeted for ubiquitination, de-ubiquitinating enzymes remove ubiquitin from p53 (Fig. 3). It is well known that p53 is a short-lived protein whose levels are low in normal cells and whose stability is tightly regulated through MDM2-mediated ubiquitination.^320,321^

Abundant evidence suggests that the de-ubiquitinase herpesvirus-associated ubiquitin-specific protease (HAUSP, also known as USP7) plays a critical role in stabilizing p53, even in the presence of excess MDM2, and that it activates p53-dependent cell arrest and apoptosis.^322,323^ HAUSP was also shown to form a complex with MDM2 and p53. The TRAF-like domain of HAUSP is regarded as the necessary region to bind to p53, and HAUSP interacts with MDM2 both in vivo and in vitro.^324,325^

In addition to de-ubiquitinating p53, HAUSP also controls MDM2 de-ubiquitination. Thus, HAUSP-mediated de-ubiquitination can bring about increased levels of MDM2 that then accelerate p53 degradation to directly reduce the level of p53. In normal cells, MDM2 is the preferential HAUSP substrate; thus, p53 accumulates due to MDM2 destabilization.^326^ In stressed cells, ATM is activated by DNA damage, and it then phosphorylates MDM2, which leads to a lowered affinity for HAUSP.^248^ It is an interesting finding that the effects of HAUSP on the p53 pathway depend on its concentration. Partial reductions in HAUSP levels lead to destabilization of p53, whereas more complete reductions may cause MDM2 destabilization and p53 accumulation.^327^

USP10 (ubiquitin-specific protease 10) is another de-ubiquitinase enzyme that regulates the levels of p53 by controlling p53 ubiquitination and stability.^328,329^ Unlike HAUSP, USP10 can interact only with p53, and not with MDM2. Moreover, USP10 is mainly localized in the cytosol, where its function is to maintain the levels of p53 and to counteract MDM2-mediated p53 nuclear export under normal conditions.^330^ Upon DNA damage, USP10 is phosphorylated by ATM, after which it is re-localized to the nucleus where p53 de-ubiquitination occurs, which is the reverse of the function of residual MDM2, which ubiquitinates p53.^329,330^ As USP10 plays an anti-cancer role by regulating the nuclear output and degradation of p53 induced by MDM2, down regulating DUBs may have an impact on cancer and other hypoxia related diseases.^331^

Ovarian tumor domain-containing Ub aldehyde-binding protein 1 (Otub1), DUB from the OTU-domain containing protease family, directly suppresses MDM2-mediated p53 ubiquitination in cells and in vitro.^332^ However, Otub1 decreases p53 ubiquitination, stabilizing and activating p53 in cells via inhibition of UbcH5, a cognate ubiquitin-conjugating enzyme of MDM2.^333^ Thus, Otub1 mediates p53 ubiquitination in cells independently of its de-ubiquitinating enzymatic activity.^194,332,334,335^ Furthermore, Otub1 plays a crucial role in the stability and activity of p53 after DNA damage, because Otub1 can inhibit DNA damage-induced chromatin ubiquitination and slow down DNA repair.^336^ In conclusion, Otub1 regulates the p53-MDM2 loop as a potential inhibitor of the E2 enzyme.

The ubiquitin-specific protease 2 (USP2) has two isoforms formed by alternative splicing, USP2a and USP2b.^337^ USP2a is a de-ubiquitinating enzyme that regulates the p53 pathway by interacting with and ubiquitinating MDM2 in vivo.^338^ USP2a can directly de-ubiquitinate MDM2, but not reverse MDM2-mediated ubiquitination of p53. Overexpression of USP2a causes an increase in the MDM2 protein level and accelerates the degradation of p53. Knock down of USP2a results in increased p53 protein accumulation and activation of its target genes.^338^ Thus, USP2a was identified as an important regulator of the MDM2/p53 pathway, which is important for repressing p53 activity in vivo.

The DUB ubiquitin-specific protease 24, USP24, is a 2620-amino-acid ubiquitin-specific protease, containing several conserved domains: a UBA domain, a UBL domain and a USP domain.^339^ USP24 is a DUB that increases p53 stability and activity. USP24 directly de-ubiquitinates p53 in response to DNA damage, as well as in unstressed cells.^339^ Therefore, USP24 plays a crucial role in the apoptosis pathway by maintaining p53 activation after DNA damaged.^150^ Furthermore, the USP24 level is increased by DNA damaging agents, and it plays a crucial role in maintaining genome stability.^340^

Ubiquitin-specific peptidase 29 (USP29) deconjugates ubiquitin from p53 and stabilizes p53.^341^ USP29 is activated by the far upstream element binding protein (FBP) and reverses MDM2-directed p53 ubiquitination to protect p53 from degradation. Furthermore, USP29 could stabilize p53 in an alternative mechanism via recognizing p38/AIMP2 (JTV1) pro-apoptotic potential.^341^ As a pro-apoptotic stabilizer of p53, USP29 expression is restricted in most tissues and cells through DNA methylation or repressive chromatin compaction.^342^

USP22 was initially regarded as part of an 11 gene “death from cancer signature”, which referred to tumors with a cancer stem cell phenotype.^343,344^ USP22 is a positive regulator of the NAD-dependent histone deacetylase Sirt1. USP22 mediates stabilization of Sirt1 by interacting and removing poly-ubiquitin chains previously conjugated to Sirt1. Sirt1 negatively regulates p53 transcriptional activity to inhibit cell apoptosis. Therefore, USP22 stabilizes Sirt1, leading to suppression of p53-meditated functions.^345^

In the past decade, DUBs have become an attractive target for cancer treatment for their actions are involved in many diseases such as cancer. The knowledge in the field of DUB and E3 ligase demands further exploration which may benefit to future therapies.^331^ To summarize, ubiquitination and degradation processes have a profound effect on the activity of p53. Similarly, a series of molecules are involved in de-ubiquitination, which ensures that p53 activity is strictly controlled (summarized in Table 3) (Fig. 3).

**Table 3:** Deubiquitinases and ubiquitin-like proteins that impact on the p53 pathway

De-ubiquitinase /Ubiquitinase	Target	Function
De-ubiquitinase HAUSP/USP7 USP2a USP10 Otub1 USP24 USP29 USP22 Ubiquitinase Pirh2 COP1 ARF-BP1 Ubc13 Synoviolin CARP1 Trim24 Topors UBE4B p300/CBP	p53/MDM2/MdmX p53/MDM2 p53 MDM2 p53 p53 Sirt1 p53 p53 p53 p53 p53 p53 p53 p53 p53/MDM2 p53	Stabilization^322,325,504^ Stabilization^338,505^ Stabilization^330,506^ Stabilization^332,507^ Stabilization^339,340^ Stabilization^341^ Stabilization^345^ Proteasome degradation Transactivation^279,280^ Proteasome degradation^284,287^ Proteasome degradation^289,508^ Proteasome degradation^315^ Proteasome degradation^300,304^ Proteasome degradation^509^ Proteasome degradation^295,297^ Proteasome degradation^301,510^ Proteasome degradation^216,308,511^ Transactivation^216^

In addition, DUBs is engaged in ubiquitin precursors processing, ubiquitin recycling, and ubiquitin chains editing.^346^ Thus, it is not surprising that inappropriate activity of DUBs directly or indirectly causes many diseases, including cancer, and affects many signaling pathways. Therefore, the study of p53 related DUB inhibitors and drug modification has become an important study focus in the world, such as USP10 inhibitor Spautin.^331^

### Cross talk between post-translational modifications on p53 following DNA damage

**Fig. 5:**
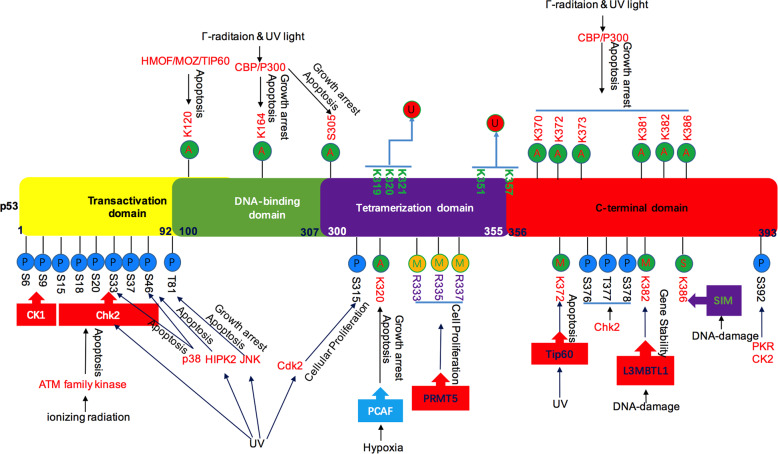
p53 structural domains and sites for post-transcriptional modifications. More than 36 amino acids of p53 are reported to be modified. The major sites of p53 post-transcriptional modification are shown with the corresponding main modifying enzymes. The modifications directly responsible for the listed effects are shown. M methylation, A acetylation, U ubiquitination, S sumoylation, P phosphorylation. CK1 casein kinase 1, Chk2 checkpoint kinase 2, ATM ataxia telangiectasia, mutated, hMOF human males absent on the first, MOZ monocytic leukemia zinc-finger protein, TIP60 HIV1-TAT interactive protein, HIPK2 homeodomain interacting protein kinase-2, JNK c-Jun amino terminal kinase, CDK2 cell cycle control regulator cyclin-dependent kinase-2, PCAF p300/CBP-associated factor, PRMT protein arginine methyltransferase, L3MBTL1 lethal 3 malignant brain tumor-like 1, SIM mortality information system, PKR double-stranded RNA-dependent protein kinase

p53 is a key mediator of cellular responses to numerous types of cellular stresses, such as DNA damage. The C terminus of p53 (positions K370, K372, K373, K381, K382, and K386) can be modified by both acetylation and ubiquitination (Fig. 5). Acetylation of p53 interrupts the interaction between p53 and MDM2 by inhibiting the recruitment of MDM2 to the p53 promoter resulting in p53 activation independent of its phosphorylation status.^186^ After DNA damage, N-terminal phosphorylation of p53 promotes the interaction of p53 with p300/CBP or PCAF and, subsequently, leads to the acetylation of the C-terminal K382 or K320 residues to active the DNA-binding activity of p53. However, repressive K382 methylation prevents acetylation by CBP/p300 at this same site, and the level of methylation at K382 decreased upon DNA damage, counteracting its inhibitory effect and promoting CBP/p300-dependent acetylation of K382.^206^ Thus, the interplay between p53 methylation, and phosphorylation, as well as acetylation, demonstrates a mechanism for modulating p53 transcriptional activity upon stress. Notably, phosphorylation at S46 and acetylation at K120 are crucial modifications for switching on p53’s pro-apoptotic function, which enables tumor cells to be removed.^347^ In short, methylation occurs at the C-terminal K370. K372 and K382 residues can also be ubiquitinated and acetylated, and p53 activity can be increased or inhibited depending on the modification site and modification mode. Normally, lysine methylation occurs upon DNA damage and then accelerates or prevents the successive acetylation of other residues^158^ (Fig. 5). Moreover, ubiquitination and deacetylation quickly weaken p53 expression and function. Therefore, cells can re-enter the cell cycle by escaping from p53-mediated cell cycle arrest.^347–349^ Collectively, these data suggest that the post-translational of p53 at different sites has different regulatory effects on the transcriptional activity of p53 through different mechanisms.

### Complex post-translational modifications on p53 in tumor tissues

Furthermore, as many as 150 different PTMs have been identified on p53, suggesting that the mechanisms of p53 post-transcriptional regulation are highly complex in normal and tumor tissues.^350^ Methylation of lysine and arginine were normally regarded as a reversible mechanism that modulate p53 function. The C terminus of p53 might function as a major site where single modifications occur, and where the K-to-R mutations occur. The variety of modifications and the many modification sites make it very complicated to elucidate the mechanisms by which p53 function is fine-tuned.^351^ Therefore, extremely careful research using mouse models is needed to study tissue-specific and cell-type-specific changes in p53 function that result from changes in post-translational modifications. Currently, it is not completely clear whether there are other sites, new functions, or new mechanisms that take part in the post-transcriptional modification of p53. Moreover, it is unclear how the modification of p53 influences cells and tissue in a tumor-specific manner. Further studies of specific tumors may help to identify additional attractive targets for radiotherapy and chemotherapy.^34,352^

### Post-translational modifications—modifying the p53 function in mice model

p53^S18A^ knock-in mice, in which serine 18 was mutated to a non-phosphorylatable alanine.^353^ Phosphorylation of p53 serine 18 does not affect the stability of p53 protein, but contributes to the activation of p53 target genes, thus participating in p53-dependent apoptosis and delayed tumor suppression.^354^ p53^S23A^ knock-in mice, in which serine 23 was mutated to a non-phosphorylatable alanine. There are data indicate that serine 23 phosphorylation response to DNA damage contributes to the stabilization of p53 protein and cell type dependence of p53-dependent apoptosis, as well as to inhibit the occurrence of B-cell lymphoma.^355^ p53^HupKIS46A^, a HupKI mouse strain with serine 46 mutated to non-phosphorylatable alanine, was established to study the role of serine 46 phosphorylation in vivo.^356,357^ This residue plays a major role in p53-mediated apoptosis. p53^S389A^ knock-in mice was produced and studies have shown that serine 389 phosphorylation selectively promotes apoptosis and tumor suppression under ultraviolet irradiation.^358^ p53^S312A^ knock-in mice was generated and at this site, ES cells play a key role in the Nanog inhibition and ES cell differentiation, suggesting that serine 315 phosphorylation also plays a role in stem cells.^359^ Mouse p53 C-terminal contains many lysine residues (K367, K369, K370, K378, K379, K383, and K384), which can be modified by ubiquitination, acetylation, diacylation, sumoyation, or methylation. Two knock-in mouse strains address the importance of these residues by mutating all C-terminal lysine into arginine to block any modification of these residues. The “p53^6KR^” knock-in mouse strain carries six C-terminal lysine mutations (K367R, K369R, K370R, K378R, K379R, and K383R), while the second “p53^7KR^” mouse strain has seven mutations, including the above mutation and one mutation at lysine 384 (K384R), which is a non-conservative sequence in human genes.^359,360^ To clarify the role of a single lysine, some studies have examined the effects of altering a single lysine, such as a murine strain, p53^K317R^ in lysine knock-in mice, causing acetylation loss on the residue, and acetylation at lysine 317 negatively regulates p53 transcriptional activity.^361^ The Asn-to-Ser substitution p53 (p53N236S) knock in mice model promotes female embryos neural tube defects.^362^

The mouse models mentioned above are summarized in Table 4 to provide insight into how post-translational modifications of p53 is linked to its function. PTM mutant mice may exhibit positive or negative regulation of p53 activity.^363,364^ Thus, future research will further understand the specific role of each PTM and how modification can be used as a therapeutic target for cancer. Thus, PTM site mutant mice may exhibit positive or negative regulation of p53 activity. Future research will understand the specific role of each PTM and show how modifications can be used as a therapeutic target for cancer.

**Table 4:** p53 modifications in vivo for p53 as a tumor suppressor protein

Mouse model	Function	p53 modifications in vivo
p53^S18A^ knock-in mice p53^S23A^ knock-in mice p53^HupKIS46A^knock-in mice p53^S389A^ knock-in mice p53^S312A^ knock-in mice p53^6KR^ knock-in mice	p53-dependent apoptosis and tumor suppression Stabilization of p53 protein and cell type dependence of p53-dependent apoptosis p53-mediated apoptosis Selectively promotes apoptosis and tumor suppression under ultraviolet irradiation Stem cells DNA damage	Serine18 mutated to non-phosphorylatable alanine^353,512,513^ Serine18 mutated to non-phosphorylatable alanine^355,513,514^ A HupKI mouse strain with serine 46 mutated to non-phosphorylatable alanine^356,357^ Serine389 mutated to non-phosphorylatable alanine^358,515^ Serine312 mutated to non-phosphorylatable alanine^359,364^ Six C-terminal lysine mutations (K367R, K369R, K370R, K378R, K379R, and K383R)^516^
p53^7KR^ knock-in mice	DNA damage	Seven C-terminal lysine mutations (K367R, K369R, K370R, K378R, K379R, K383R, and K384R)^359,360^
p53^K317R^ knock-in mice	Negatively regulates p53 transcriptional activity	Lysine317 mutated to non-acetylated arginine^361^
p53^N236S^ knock-in mice	Female embryos neural tube defects	Asparagine236 substitute to serine^362,517^
p53^K120R^ knock-in mice	mRNA decay	Lysine120 substitute to arginine^518^

### Therapeutic strategies to restore wild-type activity of mutant p53

A variety of strategies for tumor expressing p53 mutant, for p53 having many different mutations. Wild-type p53 in tumor cell is an effective activator of apoptosis and senescence, making the reactivation of certain wild-type functions of mutant p53 (usually overexpressed in cancer) a promising therapeutic pathway. Interestingly, the wild-type loss of function caused by some unstable tumor-derived mutations can be remedied by another point mutations that help stabilize the integration of the p53 protein, suggesting that the change of structure is reversible.^365^ Small molecules such as PhiKan083 and PK7088 bind to a site of p53 and form the Y220C mutant, which will stabilize this mutant and increase the level of wild-type p53.^366^ Further, other molecules bind to a variety of mutant p53 proteins and interact with DNA binding domains to promote the correct folding of the mutant protein and the recovery of p53 function, PRIMA-1, PRIMA-met/APR-246, CP-31398, and SCH29074 for example.^367–369^ Wild-type p53 needs to bind to Zn (2+) to fold correctly, while R175H p53 mutant is damaged in zinc binding.^370,371^ While the addition of zinc to the conformational mutants G245C and G245D p53 partially recover the wild-type constellation.^372,373^ Therefore, the potential of zinc to restore wild-type folding has been discovered, and this method has been proved to recover chemosensitivity to anticancer drugs in cells which express mutant p53 protein.^374^ In addition, it was found that NSC31926, a thiourea metal chelator, can restore p53 wild-type function in many different cell lines expressing p53 mutants, possibly by enhancing the bioavailability of zinc to p53 mutants.^375^

Although there are components targeted to mutant p53, many of them also interact and inhibit p53 family proteins, p63 and p73. A small component called RETRA, discovered by chance in a screening of a drug used to determine stable wild-type p53, is thought to disrupt the p73 mutant with p53 interaction. RETRA induced p73 release led to activation the targeted gene for p73, suppressed tumor cell survival and inhibited xenograft tumor growth^376^ (Table 2).

### Complexity of p53 regulation: post-translational modifications and cross talk with each other

The scope of the post-translational modifications of p53 is deeper and more complex than previously reported. These modifications engaged in p53 level, activity, protein–protein interaction, subcellular localization, and crosstalk from other signaling pathways. The extensive list of p53 post-translational modifications suggest that there is a dazzling arrangement that may exist in p53, therefore, for its functional status at any given time and in any particular biological context. Due to the complexity of those PTMs, future analysis will focus on some certain amino acid sites of p53 and cross talk of PTMs with good characteristics.

### PTEN: multiple roles in human cancers

Tumor suppressor, PTEN, a phosphatidylinositol 3,4,5-triphosphate (PIP3) lipid phosphatase, is frequently inactivated in cancer by mutation, epigenetic silencing, or PTMs.^377,378^ PTEN plays an important role in regulating cell growth, apoptosis, mobility, proliferation, signal transduction and other key cell processes.^379^ PTEN is affected by phosphorylation, ubiquitination, acetylation, SUMOylation, and oxidation of active sites.^380,381^ Some post-translational modifications can lead to the deactivation of PTEN function rather than the goal of PTEN gene integrity.^382–384^ Post-translational modification can dynamically change activity and function of PTEN and abnormal in the post-translational modulation of PTEN brings about cell proliferation, migration, and adhesion, which are related to the occurrence, development and metastasis of cancer.^385,386^

### PTEN phosphorylation is a new mechanism of PTEN inactivation that plays an important role in tumorigenesis

**Fig. 6:**
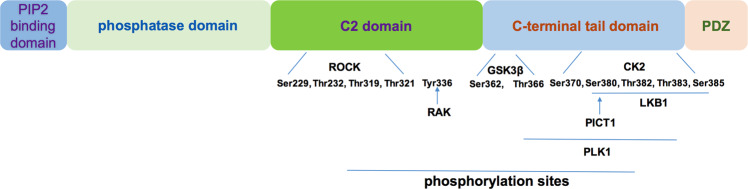
PTEN structural domains and sites for phosphorylation. PTEN structured domains include the PIP2 binding domain, phosphatase domain, two C-terminal domains, and PDZ domain. PIP2 phosphatidylinositol diphosphate, PDZ post-synaptic95, disks large, zonula occludens, CK2 casein kinase 2, GSK3β glycogen synthase kinase-3β, LKB1 liver kinase B1, PICT1 protein interacting with carboxyl terminus 1, PLK1 polo-like kinase 1, PTEN phosphatase and tensin homolog, ROCK rhoA-associated protein kinase, Ser serine, Thr threonine, Tyr tyrosine

PTEN is a double lipid and protein phosphatase that works as a tumor suppressor through several AKT-dependent and independent pathways.^387^ PTEN protein has 403 amino acids and contains five crystal domains. One N-terminal (PIP2) binding domain, one N-terminal phosphatase domain, one C2 domain, one C-terminal tail domain rich in proline (P), glutamic acid (E), serine (S), and threonine (T) and various phosphorylation sites and one PDZ interaction region (Fig. 6).^388^ PTEN has six sites of phosphorylation, which are related to the regulation of tumor suppressive function, stability, and subcellular regionalization.^389^ Phosphorylation of Ser380, Thr382, Thr383, and Ser385 which are sites of PTEN in its C-tail region results in the intramolecular binding of C-terminal tail of PTEN with the rest of the PTEN body, which leads to the blocking/inactive conformation of PTEN, thus reducing the catalytic activity and membrane binding.^390^ Each of the four sites helps to stabilize the closed conformation of PTEN, and at least three sites are needed to make up with the full effect of tetraphosphate PTEN, which imply that the dynamic step-by-step closure of PTEN conformation may occur by modifying only one subset of Ser/Thr residues, which in turn may lead to the sliding scale of cell signaling effects.^391^

Under the treatment of ionizing radiation (IR), the phosphorylation of PTEN at 240 sites facilitates the interaction between pY240-PTEN and Ki-67, which promotes the recruitment of RAD51 to accelerate DNA repair.^392^ In glioblastoma (GBM) preclinical model, blocking Y240 phosphorylation can enhance radio sensitivity and prolong survival and Y240F-PTEN knock in mice showed radio sensitivity. FGFR-regulated pY240-PTEN is the key mechanism of anti-radiation therapy and an effective target to improve the efficacy of radiotherapy.^392,393^ E3 ubiquitin ligase Parkin mediates ubiquitination of many substrate proteins, leading to proteasome degradation. Parkin directly binds with epidermal growth factor receptor (EGFR) and promotes the ubiquitination of EGFR, leading to the decrease of activation of PI3K/AKT signal induced by EGFR, and in turn Parkin depletion promoted the inhibition of PTEN by S-nitrosylation and ubiquitination, which imply that PTEN involved in Parkin depleted PI3K/AKT-mediated cellular survival.^394^

Casein kinase 2 (CK2) interacts with PTEN physically,^395^ can phosphorylate PTEN on Thr366, Ser370, Ser380, Thr382, Thr383, and Ser385 (Fig. 6).^395–397^ The phosphorylation of PTEN by protein kinase CK2 promotes the stabilization of PTEN protein and the associated inactivation of PTEN function.^398^ Post-translational inactivation of PTEN mediated by CK2 is related to the over-activation of PI3K/AKT pathway, which is a common event in adult B-cell acute lymphoblastic leukemia, suggesting that inhibition of CK2-regulated PTEN may be an effective and novel therapeutic tool for this malignant tumor.^399^

Ser370, Ser380, Thr382, Thr383, and Ser385 of PTEN can be phosphorylated by liver kinase (LKB1), resulting in its inactivation.^400–402^ Using the conditional gene knockout alleles of LKB1 and PTEN, the inactivation of the dual alleles of the two tumor suppressor factors in the lung resulted in the pure squamous cell phenotype of lung tumors.^403^ Glycogen synthase kinase 3β (GSK3β) also play a synergistic role in PTEN phosphorylation with CK2.^396^ R280T mutation of p53 mediates the proliferation of human glioma cells associated with GSK-3β/PTEN pathway.^404^ Moreover, rhoA-associated protein kinase (ROCK) can inhibit PTEN after phosphorylation of Ser229, Thr232, Thr319, and Thr321, and then transfer it to the membrane. ROCK1 is a physiological regulator of PTEN. Its function is to inhibit excessive recruitment of macrophages and neutrophils in response to acute inflammation.^405^ Rak is a tyrosine kinase that interacts with PTEN and phosphorylates it on Tyr336 and plays a real role of tumor suppressor gene by regulating the stability and function of PTEN protein in Breast cancer.^406^ Furthermore, polo-like kinase 1 (PLK1) phosphorylated Ser-380, Thr-382, and Thr-383 of PTEN which are a cluster of residues regulating the stability of PTEN and the phosphorylation of PTEN was associated with the accumulation of it on chromatin and regulated cell cycle.^407^ Protein interacting with the carboxy terminus-1 (PICT1) was able to bind to PTEN and phosphorylated Ser-380 which is required for stability of PTEN and its mediated cervical carcinoma.^408^

In conclusion, phosphorylation of PTEN have potential to restore or enhance PTEN activity, thereby inhibiting cancer cell proliferation and resistance to chemotherapy drugs.

### Monoubiquitination of PTEN promotes nuclear localization, and polyubiquitination leads to proteasome degradation in cytosol, resulting in loss of tumor suppressive activity of PTEN

Neuronal precursor cell-expressed developmentally downregulated-4-1 (Nedd4-1), is the first considered E3 ligase for PTEN ubiquitination. NEDD4-1 can mono-ubiquitinate PTEN, which is related to nuclear shuttle, genomic stability and cell cycle arrest.^409^ NEDD4-1 also can promote polyubiquitylation of PTEN, which accelerate proteasome degradation of PTEN.^410^

In addition, X-linked apoptotic inhibitors and E3 ubiquitin ligase WW domain (WWP2) is also thought to interacts with and ubiquitinate PTEN, which regulates PTEN degradation via ubiquitination pathway.^411,412^ It is reported that WWP2 mediates cellular apoptosis, which is a necessary condition for tumorigenesis.^413^ Therefore, WWP2 might play a crucial role in the survival of cancer cells in a PTEN dependent way.^414^ Both gene ablation and drug inhibition of WWP1 can activate PTEN and release tumor suppressive activity. WWP1 is a direct MYC (MYC proto-oncogene) target gene that is key for MYC-promoted tumorigenesis. Indole-3-methanol, a compound discovered in cruciferous vegetables, as a natural and effective WWP1 inhibitor.^415^ Further, linc02023 specifically interacts PTEN, and inhibits its interaction and ubiquitination with PTEN through WWP2, making it stable and inhibiting its downstream expression, suggests that linc02023 may be a new therapeutic target by restoring the antitumor activity of PTEN.^416^ Therefore, it is a potential therapeutic strategy for the prevention and treatment of cancer through via activation of PTEN.

### PTEN-opathies: molecular targeted therapy

The changes of PI3K/AKT/mTOR signaling pathway in PTEN mutant cancer patiens indicated that PI3K, AKT or mTOR are target for molecular therapy.^417^ PTEN hamartoma tumor syndrome (PHTS) is caused by pathogenic PTEN mutation in germline. mTORC1 inhibitor rapamycin reduces symptoms and excessive growth in PHTS patients.^418–420^ In fact, rapamycin has been tested in patients with PHTS in phase II open clinical trials.^421^ In addition, the upstream proteins of PTEN signaling pathway, PI3K and AKT for example, can also be used as drug inhibition candidates for PTEN mutant patients. Drug Wortmannin and AQR 092 are target PI3K and AKT, respectively^422,423^ (Table 2). Therefore, inhibitors of AKT and PIK3CA are used in Poteus and Proteus-like syndromes and PIK3CA related over growth disorders.^422,424,425^ In addition, constitutional PTEN pathway dysfunction theoretically requires some kind of chronic treatment program. However, lifelong suppression of mTOR and PIK3CA may not be executive due to immunosuppression, the destruction of systemic glucose homeostasis and the important role of PTEN pathway in normal tissue and organ development.^425–427^ Another important warning for molecular targeting of PI3K/AKT/mTOR pathway is that feedback induction of collateral carcinogenic signaling pathway leads to drug resistance. This has led to the study of combination therapies that, in theory, can effectively target excessive growth signals without losing control of feedback. In fact, mTORC1 inhibition has been demonstrated effectively bring about feedback activation of upstream signaling components.^428^ Although most treatment strategies aim to reduce the downstream carcinogenic signal caused by PTEN dysfunction, strategies to improve PTEN level and/or activity also demonstrate promising treatment models. This is especially relevant for cell infiltration of PTEN-L, the first found isoform delegates a long PTEN protein called PTEN long (PTEN-L), which will theoretically allow the recovery of PTEN levels in the context of insufficient PTEN haplotypes.^429^ Another possible way is to restore or even enhance the function of PTEN by editing the mutated PTEN allele. Although gene editing has brought many challenges, including miss target effect and induction of adaptive immune response, recent progress shows hope in reducing these results.^188,430^ There is no doubt that gene editing will be very challenging in the reproductive environment of the whole organism.^430–432^

PI3K/AKT/mTOR pathway is also a crucial pathway of immune regulation.^433,434^ Since PTEN is the main controller of this pathway, it is not surprising that the destruction of PTEN leads to immune disorders. The latter is closely related to the occurrence of cancer. Immune surveillance, immune recognition evasion and the microenvironment of chronic inflammation are the main immune characteristics of cancer.^435^ In addition, activation of PI3K/AKT/mTOR pathway has been discovered to regulate the response of immunotherapy. The loss of PTEN in sporadic environment has always been related to the drug resistance of anti PD-1 in the treatment of melanoma. Recently, PTEN has been used in the case study of metastatic uterine leiomyosarcoma.^436^ Interestingly, the PI3K/AKT/mTOR pathway activated has been demonstrated to drive the expression of PD-1/PD-1L in some solid tumors, leading to immune tolerance.^437–439^

### Significance of the Rb–p53–PTEN network to cancer

Rb is the most common mutation gene in childhood cancer retinoblastoma, and its deletion leads to E2F transcription factor induced proliferation related genes.^440,441^ However, the increase of E2F level after pRb loss can also activate apoptosis associated genes, as a protective mechanism against sudden tumor. Further, the accumulation and apoptosis induced p53 are considered to be the main mechanism to reduce the abnormal high level of E2F activity.^442^ Thus, PTEN/PI3K/AKT pathway on Rb/E2F apoptosis suppression may supply a potential therapy for retinoblastoma.

**Fig. 7:**
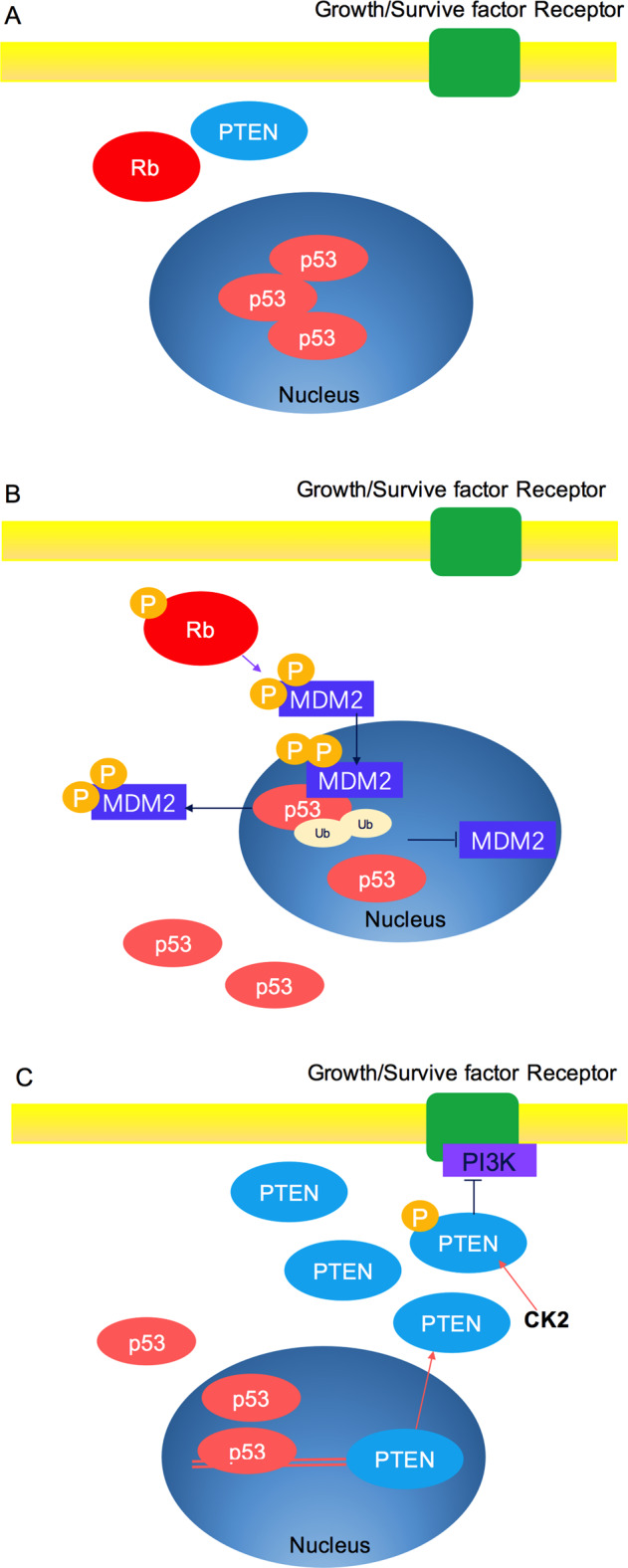
Response of cell stress on regulation of p53 function by Rb and PTEN. **a** The response of cells to stress is phosphorylation of Rb, and the stabilization of the p53 protein. Such stabilization readies the cell for an apoptosis. **b** One of the target genes activated by p53 is MDM2. Nuclear entry of MDM2 blocks p53 transactivation and promotes p53 degradation. **c** p53 activates the gene encoding PTEN. PTEN protein inhibits PI3K signaling and increases cellular levels of p53. Induction of PTEN by p53 could enhance p53 function and activate apoptotic response of cells

PTEN encodes a lipid phosphatase which antagonizes PI3K, and these two genes are often lost in many human cancers.^35,443^ Further, mutated PTEN are discovered in rare autosomal dominant cancer susceptibility syndromes, such as Cowden’s disease.^444^ The gene p53 deleted, point mutated and allele lost are common in most human cancers.^445,446^ p53 mutation is also related to Li Fraumeni syndrome which is susceptible to hereditary cancer.^253^ Therefore, Rb, E2F, PTEN, PI3K, AKT, and p53 are all involved in the function of cell growth, and gain or loss function of TSGs and oncogene. The abnormal network of those genes can bring about unregulated growth. PTEN can inhibit PI3K-AKT pathway that can promote the nuclear localization of MDM2 and the downregulation of p53, which may reveal the mechanism of cancer chemotherapy resistance to a certain extent.^447,448^ Cancer produces growth and survival factors that activate PI3K through autocrine or paracrine mechanisms. PI3K-kinase is a component that can be detected in many human cancers and it is associated with cell cycle arrest, inhibited apoptosis, increased tumor cells resistance to chemotherapy.^449–453^ Chemotherapy resistance stems from the following facts:

Treatment drugs could damage DNA, which promotes p53 activation. Lack of functional PTEN, or inappropriate activation of PI3K–AKT will ring from downstream target of PTEN, which will decrease p53 activity and disable cancer cells make a proper response to DNA damage. Restoration of PTEN, the development of small molecule inhibitors of PI3K and its targets, including MDM2, or elevation of p53 expression in tumor cells through gene therapy could inhibit tumor growth and sensitize refractory cancers to chemotherapy. The recovery of PTEN function and investigation of small molecule inhibitors to PI3K and its targets, covering MDM2, or the enhancement of p53 expression in tumor cells through gene therapy, can stop tumor growth and make refractory tumors sensitive to chemotherapy^35^ (Fig. 7).

## Conclusions

Under physiological conditions, tumor suppressor genes are finely regulated. These genes act as a role in the normal survival of cells by modulating the cell cycle and activating other genes engaged in the cell’s response to DNA damage, as well as inhibiting carcinogenesis, and mutation or deletion of these tumor suppressor genes may result in the deactivation of tumor suppressor, and then lead to the occurrence of malignant tumors. However, Rb deletions are almost universal in neuroendocrine prostate cancer, characterized by frequent concurrent changes in PTEN and p53. p53 mutation may also lead to poor response to androgen receptor targeted therapy of castration-resistant prostate cancer.^454^ Absent of PTEN is linked to the enhanced risk of cancer recurrence and metastasis after treatment.^455^ The loss of PTEN accelerated the medullary thyroid carcinoma induced by the loss of p53 and Rb.^456^ In high grade serous ovarian cancer, there is signaling between p53, PTEN, and Rb which contributes to tubal epithelial stem cell maintenance and the main drivers of cell transformation.^457^ In adult brain, the synergistic effect of PTEN, p53, and Rb pathway can produce high-grade astrocytoma.^68^ Inactivation of these three tumor suppressor genes was also detected in the stroma of oropharyngeal, breast, and other tumors. The mouse model demonstrated the tumor promoting effect of deletion of Rb, Pten, or p53 in fibroblasts, which transformed normal fibroblasts into cancer-related fibroblasts.^71^ The above suggests the interaction of signaling pathways managed through tumor suppressors, and those three major tumor suppressor genes interact with each other in the development and progression of these tumors, and PTMs play an important role in it.

In addition, PTM can improve the stability of complex signaling pathways through a variety of regulatory mechanisms. PTM is closely related to the occurrence, spread and metastasis of tumors; however, the underlying molecular mechanisms are still poorly understood.^47,449^ In most cancers, PTM is significantly changed, so it may become a potential target of cancer treatment. PTMs can be used as a biomarker of disease status, and its application in the assessment and monitoring of cancer disorders is a new clinical focus.^458,459^ p53 gene is now thought to encode as many as 12 different isoforms, some of which may experience PTM, suggesting that there is a great number of structural permutations possible for p53 and its function can change based on a profoundly complex variety of PTMs.^153^

**Fig. 8:**
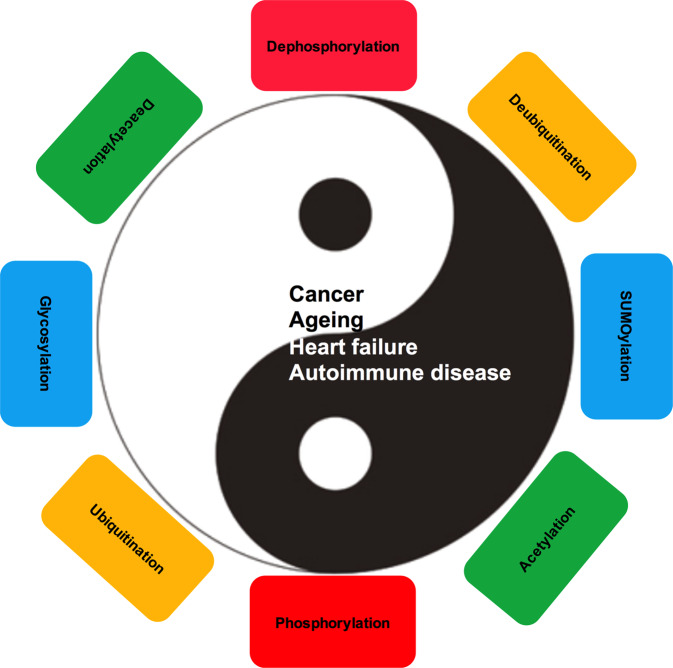
Interplay among post-translational modifications (PTMs) in the regulation of disease. Five main PTMs (phosphorylation, ubiquitination, acetylation, sumoylation and glycosylation) as well as their relative reverse processes (dephosphorylation, deubiquitination, and deacetylation) are involved in the regulation of cancer. In conclusion, the balance of cell (Yin–Yang; Yin, black; Yang, white) is crucial for maintaining cell fundamental functions, whereas dysfunction is associated with normal aging as well as with many human diseases including premature aging diseases, cancer, heart failure, autoimmune disease, and neurodegenerative disease

Dysfunctional of TSG is part of signal pathway, and the carcinogenesis is regulated by over activation of the pathway. In this case, inactivated TSG can be a therapeutic target by inhibiting the downstream associated pathways. One example is PTEN, one of the most common TSG changes in human malignancies. PTEN is inactivated with a significant proportion of mutations or deletions in a variety of cancer types, such as glioblastoma, endometrial, prostate, uterine and breast cancers, and melanoma.^15,426,460^ Post-translational modifications of TSG impact downstream targets of TSG, and can influence their functions involving in cancer, ageing, heart failure, autoimmune disease and so on (Fig. 8).^461^ The reversible processes of post-translational modification provide a complex regulatory net in the TSG pathway, including the maintenance of low p53 protein levels via ubiquitination, and p53 localization, which is related to ubiquitination, de-ubiquitination and SUMOylation. The TSG post-translational modification network may be different in different species. For example, the p53-responsive binding sites guiding apoptosis in mice do not appear to be functional in primates.^462,463^ Ubiquitination and de-ubiquitination have received much more attention.^235,464–466^ Nevertheless, many questions remain about how E3 ligases mediate p53 ubiquitination or what controls the activity of de-ubiquitinating enzymes. Future studies will most likely focus on in vivo experiments to elucidate the complexity and functions of post-translational modifications in the modulation of TSG activity. Clinical strategies may be intended to overcome chemo-resistance by inhibiting TSG degradation or other modifications.^467^ The design of TSG molecular inhibitors that target the ubiquitination pathway might be an intriguing anticancer strategy in the future.^468,469^

There are several questions to be launched. Do any other kinds of PTMs exist? Are there any other PTM enzymes not related to what have already been found? Are PTMs genuinely associated with tumor suppression or progression? If PTMs enzymes do not directly play a key role in tumor suppression or progression, then is it possible that they control one or new homeostatic mechanisms? Furthermore, given that TSG wild-type or mutant forms inhibit or promote the expression of many target genes, what role do PTMs enzymes play in these processes? Future research shows that absolute modifying factors of disease performance and related signal networks will be the most important factors to define more accurate and effective prevention and treatment strategies for individuals at risk.

Of note, few other studies have reported the role of PTMs in crosstalk of tumor suppressor genes, especially in Rb, p53, and PTEN which are more obviously affected by PTMs. Future research will be necessary to pay attention to the proteomics so that we can fully understand the role of different PTMs in regulating TSGs in cancer.
